# Foxp1 is critical for the maintenance of regulatory T-cell homeostasis and suppressive function

**DOI:** 10.1371/journal.pbio.3000270

**Published:** 2019-05-24

**Authors:** Jiazi Ren, Lei Han, Jinyi Tang, Yuanhua Liu, Xiaoxue Deng, Qiuyue Liu, Pei Hao, Xiaoming Feng, Bin Li, Hui Hu, Haikun Wang

**Affiliations:** 1 CAS Key Laboratory of Molecular Virology and Immunology, Institut Pasteur of Shanghai, Chinese Academy of Sciences, Shanghai, China; 2 University of Chinese Academy of Sciences, Beijing, China; 3 State Key Laboratory of Experimental Hematology, Institute of Hematology and Hospital of Blood Diseases, Chinese Academy of Medical Sciences and Peking Union Medical College, Tianjin, China; 4 Shanghai Institute of Immunology, Shanghai JiaoTong University School of Medicine, Shanghai, China; 5 Department of Microbiology, School of Medicine, University of Alabama at Birmingham, Birmingham, Alabama, United States of America; Children's Hospital of Philadelphia and The University of Pennsylvania School of Medicine, UNITED STATES

## Abstract

Regulatory T (Treg) cells play central roles in maintaining immune homeostasis and self-tolerance. However, the molecular mechanisms underlying Treg cell homeostasis and suppressive function are still not fully understood. Here, we report that the deletion of another P subfamily members of the forkhead box (Foxp) subfamily member Foxp1 in Treg cells led to increased numbers of activated Treg (aTreg) cells at the expense of quiescent Treg cells, and also resulted in impaired Treg suppressive function. Mice with Foxp1-deficient Treg cells developed spontaneous inflammatory disease with age; they also had more severe inflammatory disease in colitis and experimental autoimmune encephalomyelitis (EAE) models. Mechanistically, we found that Foxp1 bound to the conserved noncoding sequence 2 (CNS2) element of the Foxp3 locus and helped maintain Treg suppressive function by stabilizing the Foxp3 expression. Furthermore, we found that Foxp1 and Foxp3 coordinated the regulation of cytotoxic T-lymphocyte-associated protein 4 (CTLA-4) expression levels. Taken together, our study demonstrates that Foxp1 plays critical roles in both maintaining Treg cell quiescence during homeostasis and regulating Treg suppressive function.

## Abstract 2

The transcription factor Foxp3 has been considered as the master regulator of regulatory T cells (Tregs), but this study shows that another Foxp subfamily member, Foxp1, plays important roles in the homeostasis, stability, and suppressor function of Tregs.

## Introduction

Regulatory T (Treg) cells are a subset of CD4^+^ T cells of vital importance in the maintenance of immunological self-tolerance and T-cell homeostasis [1,2]. Like conventional CD4^+^ T cells, Treg cells in the periphery have subpopulations with different functions. Based on the expression levels of cell surface markers CD44 and CD62L, Treg cells can be divided into CD44^low^CD62L^high^ resting Treg (rTreg) and CD44^high^CD62L^low^ activated Treg (aTreg) cells [3,4]. rTreg cells are more quiescent Treg cells in the secondary lymphoid tissues, where paracrine interleukin 2 (IL-2) is required for their homeostasis, and rTreg cells have less spontaneous proliferation [3]. In contrast, aTreg cells are also present in the secondary lymphoid tissues, but they are the predominant Treg cell population in the nonlymphoid tissues [4,5]. Compared with rTreg cells, aTreg cells appear to be more potent suppressors in anti-inflammatory responses [5]. Studies have shown that transcription factors, including forkhead box O1 (Foxo1), myeloblastosis oncogene (Myb), interferon regulatory factor 4 (IRF4), the c-Rel subunit of activation of nuclear factor κB (NF-κB c-Rel), are important for aTreg differentiation and migration [4,6–8]. The transcriptional regulation of rTreg and aTreg cell homeostasis, however, is poorly understood.

Transcription factor Foxp3 has been considered the master regulator of Treg cells, indispensable for Treg cell development, maintenance, and suppressive function [9]. Loss-of-function mutations of Foxp3 are sufficient to drive lethal systemic autoimmunity both in mice and in humans due to defects in Treg cell development [10–12]. The deletion of Foxp3 in mature Treg cells leads to abnormal target gene expression and their impaired suppressive function [13]. It has also been reported that attenuation or unstable Foxp3 expression impairs the function of Treg cells [14–16], suggesting that the stringent regulation of Foxp3 expression levels is important for the maintenance of Treg cell function and identity. Studies have shown that transcription factors, such as GATA binding protein 3 (Gata3), E26 avian leukemia oncogene 1, 5′ domain (Ets-1), cyclic-AMP response element binding protein (CREB), forkhead box O (Foxo), SMAD family member 3 (Smad3), nuclear factor of activated T cells (NFAT), signal transducer and activator of transcription 5 (STAT5), runt related transcription factor 1 (Runx1), reticuloendotheliosis oncogene (c-Rel), and special AT-rich sequence binding protein 1 (Satb1), have the ability to transactivate Foxp3 expression by directly binding to the conserved noncoding sequences (CNSs) of the Foxp3 locus [17–26]. CNSs include a promoter, CNS1 and CNS2 in the first intron, CNS3 downstream of exon 1, and CNS0 in an intron of the neighboring gene 5′ of the Foxp3 locus [26,27]. The functions of CNSs have been intensively studied: CNS1 is important for the generation of Treg cells generated in the periphery (pTreg); CNS3 acts as a pioneer element, indispensable for both the differentiation of thymus-derived Treg cells (tTreg) and pTreg; and CNS2 does not affect thymic generation of Treg cells, but rather it helps maintain the stable expression of Foxp3 and protects Treg cell identity under destabilizing conditions [15,16,27]. Despite all this work, the transcriptional regulation of Foxp3 in Treg cells is not fully understood.

Studies have suggested that Foxp3 alone is not sufficient to determine the Treg cell differentiation; instead, Treg differentiation and functional maintenance need a well-orchestrated transcriptional network, including the interactions between Foxp3 and its partners [9]. A large number of Foxp3 partners have been identified as transcription-related proteins, including transcription factors: nuclear factor of activated T cells, cytoplasmic, calcineurin-dependent 2 (NFATc2), Runx1, B cell leukemia/lymphoma 11B (Bcl11b), forkhead box P1 (Foxp1), forkhead box P4 (Foxp4), Gata3, signal transducer and activator of transcription 3 (STAT3), IKAROS family zinc finger 1 (Ikaros/Ikzf1), IKAROS family zinc finger 3 (Aiolos/Ikzf3), E26 avian leukemia oncogene (Ets), and CCR4-NOT transcription complex, subunit 3 (Cnot3) [28]. Among these identified partners, Foxp1 is one of the P subfamily members of the forkhead box (FOX) transcription factor family, as is Foxp3. Our studies have revealed that Foxp1 is vital for both T-cell and B cell development and function [29–32]. In T cells, Foxp1 actively maintains naive T-cell quiescence by restraining IL-7Rα expression levels [31]. Furthermore, Foxp1 negatively regulates the differentiation and function of follicular helper T cells (T_FH_) [32]. Although it has been reported that Foxp1 can form a heterodimer with Foxp3 by the conserved leucine zipper domain [28,33], whether Foxp1 plays any role in Treg cell development and function is not known.

Here, we report that Foxp1 regulates Treg cell homeostasis and suppressive function. Loss of Foxp1 specifically in the Treg cells results in increased numbers of aTreg cells at the expense of rTreg cells. The mice with Treg-specific depletion of Foxp1 develop spontaneous inflammatory disease and are more susceptible to dextran sulfate sodium (DSS)-induced colitis and experimental autoimmune encephalomyelitis (EAE). Mechanistically, we found that Foxp1 binds to the CNS2 element of the Foxp3 locus and helps maintain the stable expression of Foxp3. The restoration of Foxp3 expression partially rescues the suppressive function of Foxp1-deficient Treg cells. Moreover, we found that Foxp1 coordinates with Foxp3 in regulating cytotoxic T-lymphocyte-associated protein 4 (CTLA-4) expression levels. Our findings demonstrate that Foxp1 is critical for Treg cell homeostasis, stability, and suppressive function.

## Results

### Foxp1 is differentially expressed in rTreg and aTreg cells

Previously, we have shown that Foxp1 has no effect on the generation of Treg cells in the thymus of *Foxp1*^*f/f*^*CD4*^*Cre*^ mice [30], in which Foxp1 is deleted at the double-positive (DP) thymocyte stage. Nevertheless, in *Foxp1*^*f/f*^*CD4*^*Cre*^ mice, Foxp1 deletion affects the activation and homeostasis of conventional CD4^+^ T cells [30], and it is not clear how this may obscure the impact of Foxp1 on Treg cells. To better define the role of Foxp1 in Treg cells, we crossed mice bearing loxP-flanked Foxp1 alleles (*Foxp1*^*f/f*^) [30,31] with *Foxp3*-YFP-*Cre* knock-in mice (*Foxp3*^*Cre*^) [34] to deplete Foxp1 specifically in Treg cells (*Foxp1*^*f/f*^*Foxp3*^*Cre*^).

**Fig 1:**
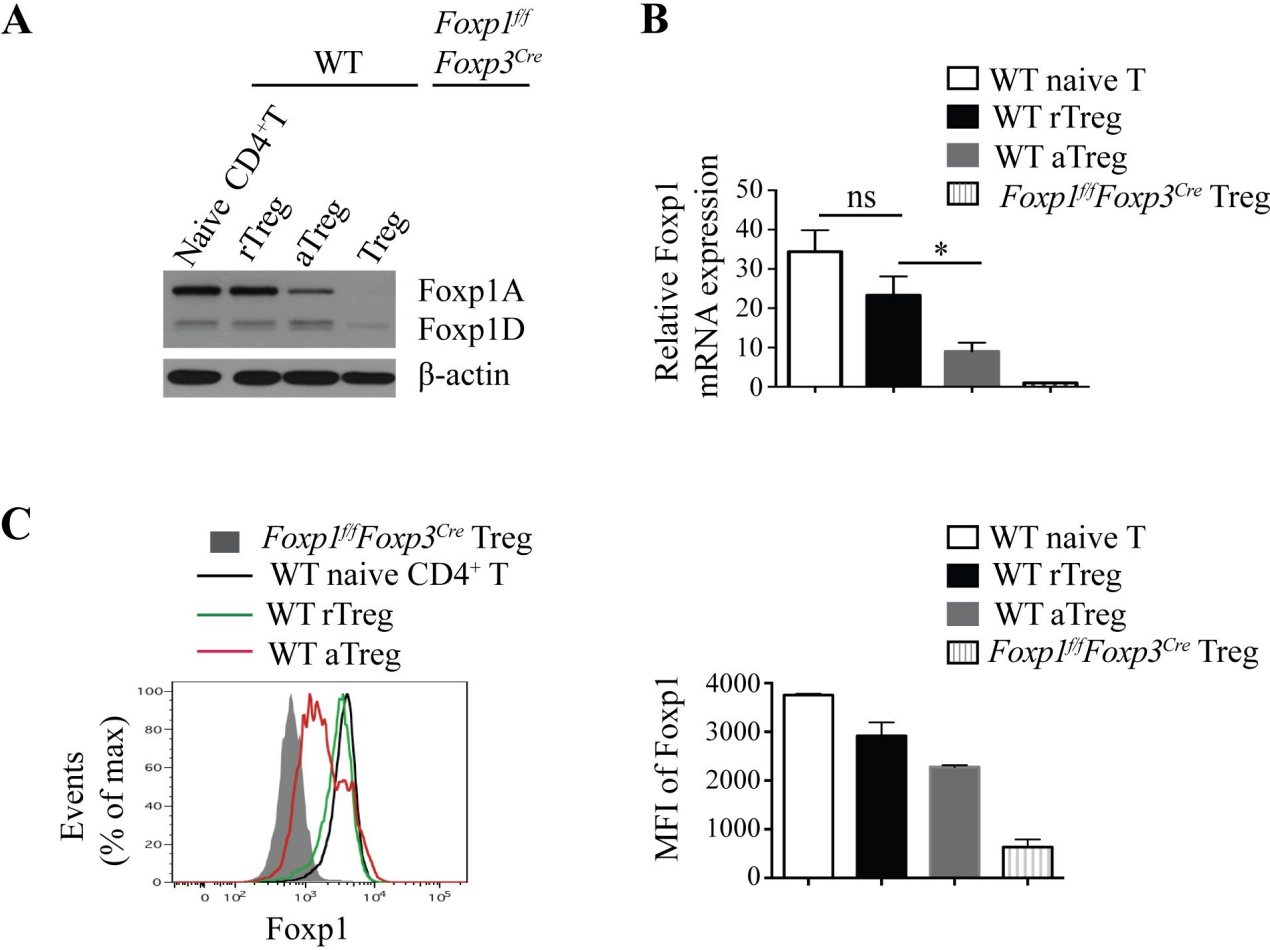
Foxp1 is differentially expressed in rTreg and aTreg cells. **(A)** Immunoblot analysis of Foxp1 expression in sorted YFP^−^CD4^+^CD44^low^CD62L^high^ naive CD4^+^ T cells (WT naive), YFP^+^CD4^+^CD44^low^CD62L^high^ rTreg cells (WT rTreg), YFP^+^CD4^+^CD44^high^CD62L^low^ aTreg cells (WT aTreg) from *Foxp3*^*Cre*^ mice, and YFP^+^ Treg cells from *Foxp1*^*f/f*^*Foxp3*^*Cre*^ mice. **(B)** Relative Foxp1 mRNA expression in sorted WT naive CD4^+^ T cells, rTreg cells, aTreg cells from *Foxp3*^*Cre*^ mice, and YFP^+^ Treg cells from *Foxp1*^*f/f*^*Foxp3*^*Cre*^ mice, *n* = 3–4. **(C)** Intracellular staining of Foxp1 in splenic WT naive CD4^+^ T cells, rTreg cells, aTreg cells in *Foxp3*^*Cre*^ mice and splenic Treg cells in *Foxp1*^*f/f*^*Foxp3*^*Cre*^ mice (left panel), and the corresponding mean fluorescence intensity (MFI) of Foxp1 (right panel) (*n* = 2). Data in **(A, C)** represent at least two independent experiments. Data in **(B)** represent four independent experiments. Data in **(B, C: right panel)** are mean ± SEM. *P < 0.05 (two-tailed Student *t* test). Data associated with this figure can be found in the supplemental data file **(S1 Data)**. aTreg, activated Treg; CD, cluster of differentiation; Foxp1, forkhead box P1; Foxp1A, Foxp1 isoform A; Foxp1D, Foxp1 isoform D; MFI, mean fluorescence intensity; rTreg, resting Treg; WT, wild-type; YFP, yellow fluorescent protein.

To understand Foxp1 functions in Treg cells, we first examined the Foxp1 expression in wild-type (WT) and *Foxp1*^*f/f*^*Foxp3*^*Cre*^ Treg cells. Given that Foxp1 is differentially expressed in conventional naive CD4^+^ T cells and activated CD4^+^ T cells [32], we reason that Foxp1 may also have different expression patterns in CD44^low^CD62L^high^ rTreg and CD44^high^CD62L^low^ aTreg (**S1 Fig**). We found that rTreg cells expressed high levels of the full-length Foxp1 isoform, Foxp1A, and low levels of the short isoform Foxp1D (**Fig 1A**), which resembles the Foxp1 expression in conventional naive CD4^+^ T cells, suggesting that Foxp1 may also play an important role in maintaining the quiescent state of rTreg cells, like its role in naive CD4^+^ T cells [31]. Surprisingly, however, we found that compared with rTreg cells, aTreg cells had lower Foxp1A expression at both the mRNA level and the protein level (**Fig 1A–1C**). This is quite different from the Foxp1 expression pattern in activated conventional CD4^+^ T cells, in which Foxp1A is expressed at a high level constitutively, and Foxp1D is greatly induced [32]. And as expected, Foxp1 was completely deleted in Treg cells from *Foxp1*^*f/f*^*Foxp3*^*Cre*^ mice (**Fig 1A–1C**). We further examined Foxp1 expression in Treg cells across various tissues, including lung, liver, and small intestine, in WT mice. Studies have shown a high proportion of aTreg cells in the small intestine [3], and, consistently, we found that among all the tissues examined, the Foxp1 expression levels were the lowest in Treg cells in the small intestine (**S2 Fig**). These results together suggest that the amount of Foxp1 protein may be important for Treg cell differentiation or function.

### Foxp1 enforces the quiescent state of Treg cells and inhibits aTreg cell differentiation

**Fig 2:**
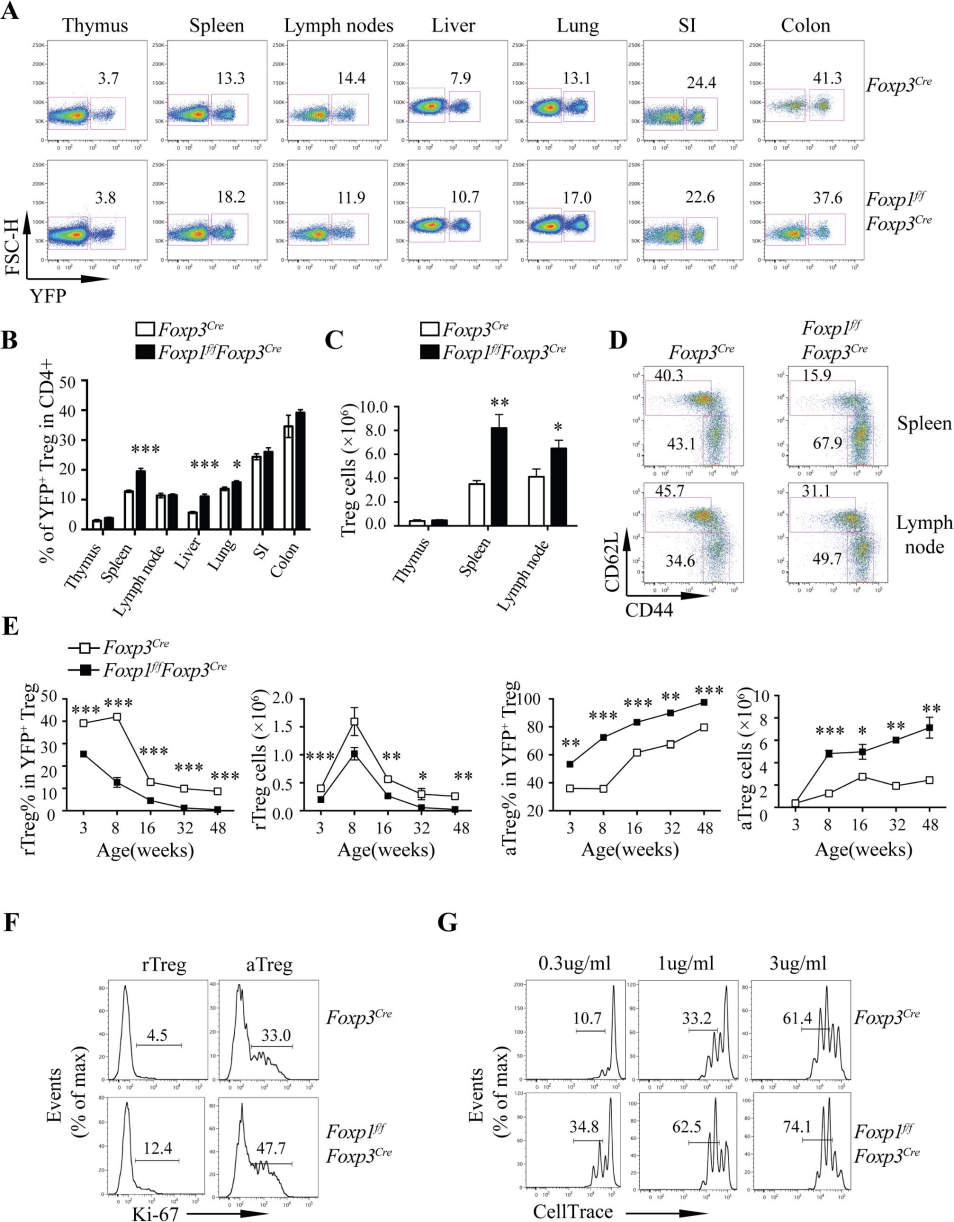
Foxp1 enforces the quiescent state of Treg cells and inhibits aTreg cell differentiation. **(A)** Flow cytometry analysis of YFP^+^ Treg cells in CD4^+^ T cells in indicated organs from 6–8-week-old sex- and age-matched *Foxp3*^*Cre*^ and *Foxp1*^*f/f*^*Foxp3*^*Cre*^ mice. Numbers adjacent to the outlined areas indicate the percentages of YFP^+^ Treg cells. **(B)** The frequencies of YFP^+^ Treg cells in CD4^+^ T cells in indicated organs from mice as in (**A**) (*n* = 3–7). **(C)** Quantification of total Treg cells in the thymuses, the spleens, and the lymph nodes from the mice as in (**A**) (*n* = 3–6). **(D)** Flow cytometry analysis of CD44 and CD62L expression in Treg cells from 6–8-week-old sex- and age-matched *Foxp3*^*Cre*^ and *Foxp1*^*f/f*^*Foxp3*^*Cre*^ mice; numbers adjacent to the outlined areas indicate the percentages of rTreg cells (CD44^low^CD62L^high^) or aTreg cells (CD44^high^CD62L^low^). **(E)** Kinetics of frequencies and numbers of rTreg and aTreg cells in *Foxp3*^*Cre*^ and *Foxp1*^*f/f*^*Foxp3*^*Cre*^ mice at different ages (*n* = 3–6). **(F)** Flow cytometry analysis of Ki-67 expression in rTreg and aTreg cells in the spleens from 8-week-old *Foxp3*^*Cre*^ and *Foxp1*^*f/f*^*Foxp3*^*Cre*^ mice; numbers above bracketed lines indicate percentages of Ki-67^+^ Treg cells. **(G)** Proliferation of rTreg cells stimulated by different concentrations of plate-bound anti-CD3/CD28 antibodies in the presence of 200 U/mL IL-2 for 3 days, as shown by the dilution of CellTrace; numbers above bracketed lines indicate percentages of Treg cells with two or more divisions. Data in **(A-E)** are representative of at least three independent experiments. Data in **(F-G)** are representative of two independent experiments. Data in (**B, C, E**) are mean ± SEM, *P < 0.05, ** *P* < 0.01, *** *P* < 0.001 (two-tailed Student *t* test). Data associated with this figure can be found in the supplemental data file **(S1 Data)**. aTreg, activated Treg; CD, cluster of differentiation; Foxp1, forkhead box P1; FSC-H, forward scatter-height; IL, interleukin; Ki-67, antigen identified by monoclonal antibody Ki 67; rTreg, resting Treg; SI, small intestine; Treg, regulatory T; YFP, yellow fluorescent protein.

To address whether Foxp1 is important for Treg cell homeostasis, we first analyzed the frequencies and cell numbers of Treg cells in the thymuses and the peripheral lymphoid and nonlymphoid organs in *Foxp1*^*f/f*^*Foxp3*^*Cre*^ mice and control *Foxp3*^*Cre*^ mice. We found that the frequencies of Treg cells in CD4^+^ T cells in a majority of the organs examined were very similar between *Foxp3*^*Cre*^ and *Foxp1*^*f/f*^*Foxp3*^*Cre*^ mice, except those in the spleen, the liver, and the lung, which were higher in *Foxp1*^*f/f*^*Foxp3*^*Cre*^ mice (**Fig 2A and 2B**). We found that the numbers of Treg cells in the spleens and the lymph nodes, but not the thymuses, of the *Foxp1*^*f/f*^*Foxp3*^*Cre*^ mice were significantly increased (**Fig 2C**), suggesting that Foxp1 deficiency has no effect on the generation or maintenance of Treg cells in the thymus, but it impairs the Treg cell homeostasis in the periphery.

Because Foxp1 is a key transcription factor that actively maintains conventional naive T-cell quiescence [31], we reasoned that Foxp1 might play important roles in maintaining rTreg cell quiescence and restricting aTreg cell differentiation. To address this question, we examined the frequencies and cell numbers of CD44^low^CD62L^high^ rTreg and CD44^high^ CD62L^low^ aTreg cells in *Foxp3*^*Cre*^ and *Foxp1*^*f/f*^*Foxp3*^*Cre*^ mice at different ages. We found that loss of Foxp1 led to significantly decreased percentages and numbers of rTreg cells, but with elevated percentages and numbers of aTreg cells in both the spleens and the lymph nodes of *Foxp1*^*f/f*^*Foxp3*^*Cre*^ mice (**Fig 2D and 2E**). Such changes were observed in *Foxp1*^*f/f*^*Foxp3*^*Cre*^ mice as early as when they were 3 weeks old (**Fig 2E**). When the mice were older than 16 weeks, *Foxp1*^*f/f*^*Foxp3*^*Cre*^ mice almost lost the rTreg cell population and compensatorily had many more aTreg cells (**Fig 2E**).

One of the pronounced characteristics of nonquiescent T cells is their enhanced proliferation capacity. To further determine whether Foxp1-deficient Treg cells lose quiescence, we examined the cell proliferation of these cells. The antigen identified by monoclonal antibody Ki 67 (Ki-67) protein is a cellular marker strictly associated with cell proliferation [35]. We found that in 3-week-old (**S3A Fig**) and 8-week-old mice (**Fig 2F**), the percentages of Ki-67^+^ cells in both rTreg and aTreg cells from *Foxp1*^*f/f*^*Foxp3*^*Cre*^ mice were increased compared with their WT counterparts, suggesting that Foxp1-deficient rTreg and aTreg cells proliferated more in vivo. We also sorted out CD44^low^CD62L^high^ rTreg cells and CD44^high^CD62L^low^ aTreg cells from *Foxp3*^*Cre*^ and *Foxp1*^*f/f*^*Foxp3*^*Cre*^ mice and labeled them with CellTrace, which tracks cell proliferation. We found that upon stimulation by different concentrations of anti-CD3/CD28 antibodies in vitro, Foxp1-deficient rTreg cells proliferated more than WT rTreg cells did (**Fig 2G**), although we did not observe obvious differences in cell proliferation between WT and Foxp1-deficient aTreg cells (**S3B Fig**). Of note, the apoptosis of rTreg and aTreg cells in *Foxp3*^*Cre*^ and *Foxp1*^*f/f*^*Foxp3*^*Cre*^ mice showed no difference (**S3C Fig**), suggesting that the higher numbers of Treg cells in *Foxp1*^*f/f*^*Foxp3*^*Cre*^ mice result from the increased Treg cell proliferation. Taken together, our results suggest that Foxp1 plays a critical role in maintaining the rTreg cell pool and negatively regulates aTreg cell differentiation.

### Foxp1 regulates rTreg cell quiescence in a cell-intrinsic manner

**Fig 3:**
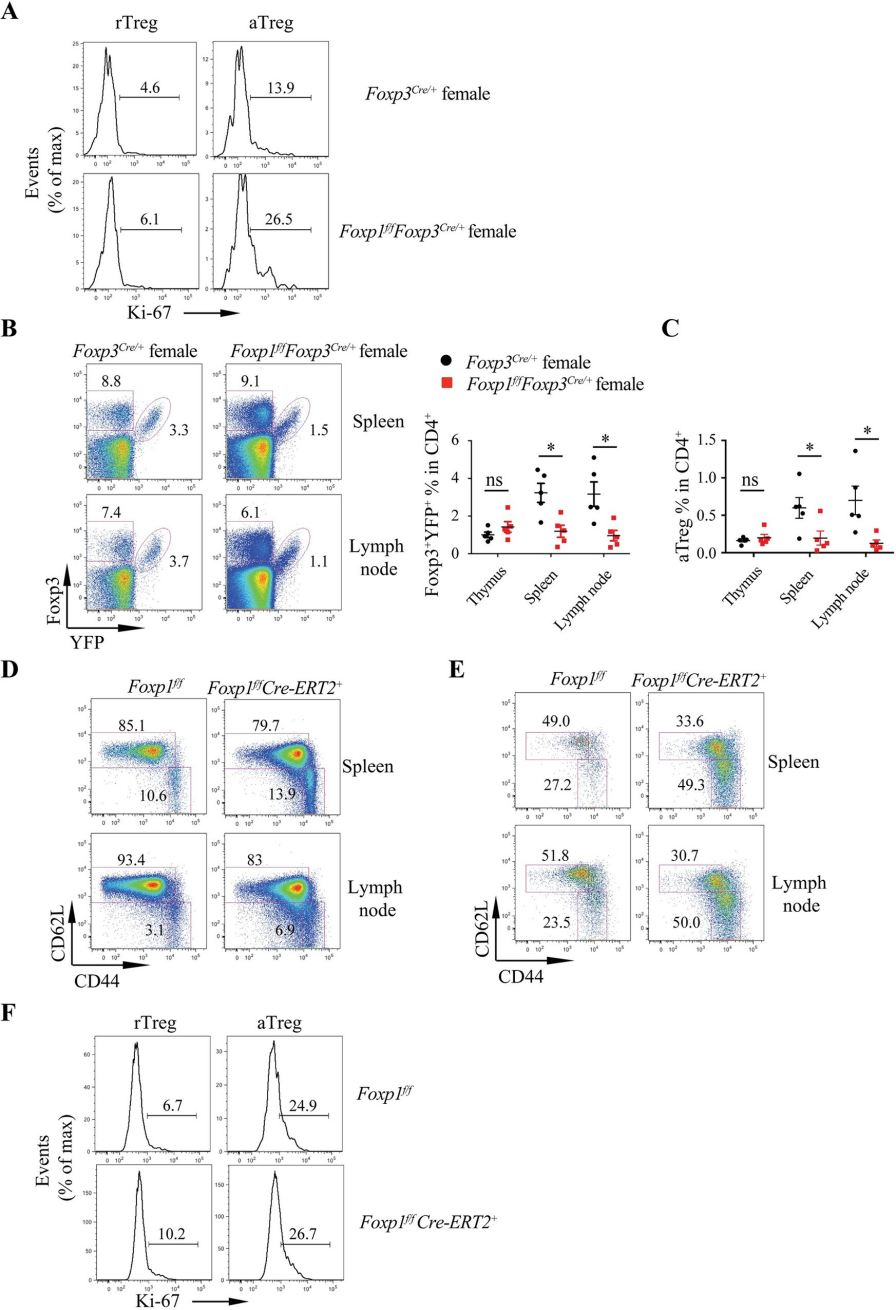
Foxp1 regulates rTreg cell quiescence in a cell-intrinsic manner. **(A)** Flow cytometry analysis of Ki-67 expression in Foxp3^+^YFP^+^ rTreg and aTreg cells in the spleens from *Foxp3*^*Cre/+*^ and *Foxp1*^*f/f*^*Foxp3*^*Cre/+*^ female mice; numbers above bracketed lines indicate the percentages of Ki-67^+^ Treg cells. **(B)** Flow cytometry analysis of Foxp3^+^YFP^+^ Treg cells from spleens and lymph nodes of *Foxp3*^*Cre/+*^ and *Foxp1*^*f/f*^*Foxp3*^*Cre/+*^ female mice (left panel), and frequencies of Foxp3^+^YFP^+^ Treg cells (right panel) (*n* = 5). **(C)** Frequencies of Foxp3^+^YFP^+^ aTreg cells in CD4^+^ T cells, *n* = 5. **(D-F)** *Foxp1*^*f/f*^ and *Foxp1*^*f/f*^*Cre-ERT2*^*+*^ mice were treated with tamoxifen, and phenotypes were analyzed at d8 after treatment. **(D)** Flow cytometry analysis of CD44 and CD62L expression in CD4^+^Foxp3^−^ conventional T cells from *Foxp1*^*f/f*^ and *Foxp1*^*f/f*^*Cre-ERT2*^*+*^ mice; numbers adjacent to the outlined areas indicate the percentages of naive cells (CD44^low^CD62L^high^) or activated cells (CD44^high^CD62L^low^). **(E)** Flow cytometry analysis of CD44 and CD62L expression in Treg cells from *Foxp1*^*f/f*^ and *Foxp1*^*f/f*^*Cre-ERT2*^*+*^ mice; numbers adjacent to the outlined areas indicate the percentages of rTreg cells (CD44^low^CD62L^high^) or aTreg cells (CD44^high^CD62L^low^). **(F)** Flow cytometry analysis of Ki-67 expression in rTreg and aTreg cells from *Foxp1*^*f/f*^ and *Foxp1*^*f/f*^*Cre-ERT2*^*+*^ mice. Numbers above bracketed lines indicate percentages of Ki-67^+^ Treg cells. Data in (**A-F**) are representative of at least two independent experiments. Data in **(B: right panel, C)** are mean ± SEM, *P < 0.05 (two-tailed Student *t* test). Data associated with this figure can be found in the supplemental data file **(S1 Data)**. aTreg, activated Treg; CD, cluster of differentiation; d, day; Foxp1, forkhead box P1; Ki-67, antigen identified by monoclonal antibody Ki 67; ns, no significance; rTreg, resting Treg; Treg, regulatory T; YFP, yellow fluorescent protein.

To address whether Foxp1 regulates rTreg cell quiescence in an intrinsic manner, we took advantage of *Foxp1*^*f/f*^*Foxp3*^*Cre/+*^ female mice, theoretically, in which around half of the Treg cells are Foxp1 sufficient, while the other half of the Treg cells are Foxp1 deficient due to the random X chromosome inactivation. Consistent with the results in *Foxp1*^*f/f*^*Foxp3*^*Cre*^ male mice, we found that the percentage of Ki-67^+^ Treg cells in *Foxp1*^*f/f*^*Foxp3*^*Cre/+*^ female mice was also higher than that in control *Foxp3*^*Cre/+*^ female mice **(Fig 3A)**, indicating that Foxp1 intrinsically regulates Treg cell proliferation. However, in *Foxp1*^*f/f*^*Foxp3*^*Cre/+*^ females, we found that Foxp1-deficient Treg cells had compromised competitive fitness in the spleens or the lymph nodes **(Fig 3B)**, with aTreg cells being almost outcompeted by WT Treg cells **(Fig 3C)**. Therefore, *Foxp1*^*f/f*^*Foxp3*^*Cre/+*^ female mice are not suitable to address whether Foxp1 intrinsically controls the balance between rTreg and aTreg. To resolve this issue, we used *Foxp1*^*f/f*^*Cre-ERT2*^*+*^ mice, in which Treg cells have normal development and function before tamoxifen treatment. We treated mice with tamoxifen and analyzed the phenotype at day (d)8. At this time point, the conventional T cells in *Foxp1*^*f/f*^*Cre-ERT2*^*+*^ mice had no obvious phenotypic alteration based on the expression of CD44 and CD62L (**Fig 3D**). However, there were more aTreg cells within the Treg cell population in *Foxp1*^*f/f*^*Cre-ERT2*^*+*^ mice **(Fig 3E)**. And consistently, acute deletion of Foxp1 led to increased Ki-67^+^ Treg cells in *Foxp1*^*f/f*^*Cre-ERT2*^*+*^ mice **(Fig 3F)**. Taken together, our data suggest that Foxp1 enforces the quiescent state of Treg cells in a cell-intrinsic manner.

### Foxp1 controls Treg suppressive function

**Fig 4:**
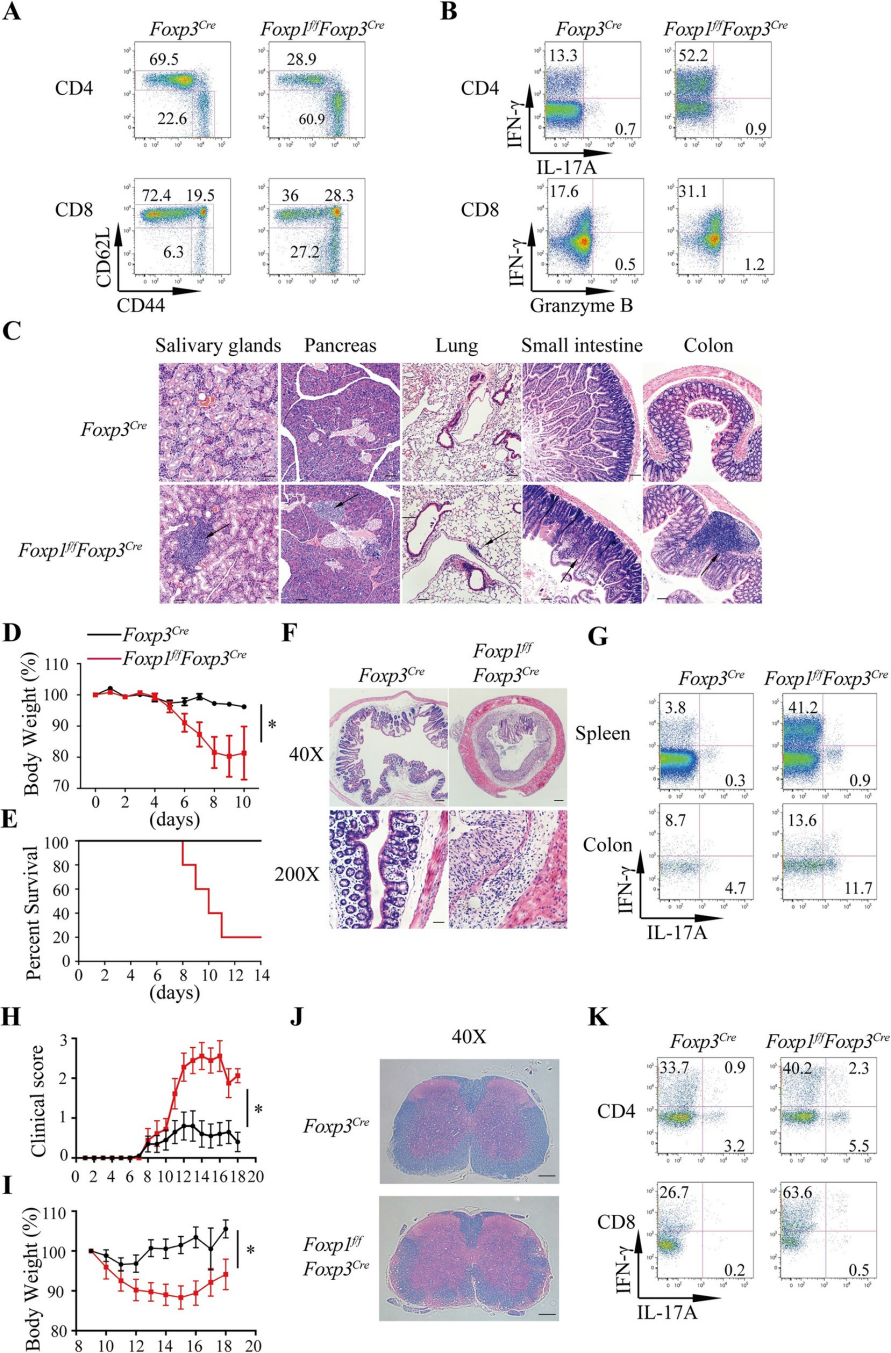
Foxp1 regulates Treg suppressive function. **(A)** Flow cytometry analysis of CD44 and CD62L expression in conventional CD4^+^ T cells (YFP^−^CD4^+^) and CD8^+^ T cells in the spleens of 28-week-old *Foxp3*^*Cre*^ and *Foxp1*^*f/f*^*Foxp3*^*Cre*^ mice; numbers adjacent to the outlined areas represent the percentages of gated cells. **(B)** Intracellular staining of cytokines in conventional CD4^+^ and CD8^+^ T cells in the spleens of 28-week-old *Foxp3*^*Cre*^ and *Foxp1*^*f/f*^*Foxp3*^*Cre*^ mice; numbers adjacent to the outlined areas represent the percentages of gated cells. **(C)** Hematoxylin and eosin staining of salivary gland, pancreas, lung, small intestine, and colon sections. The magnification is ×100. Black arrows indicate the areas of immune cell infiltration. Scale bars, 100 μm. **(D-G)** *Foxp3*^*Cre*^ and *Foxp1*^*f/f*^*Foxp3*^*Cre*^ mice (*n* = 6) were treated with 2.5% DSS, and the severity of colitis in mice was evaluated by loss of body weight **(D)**, survival curve at indicated time points **(E)**, the representative hematoxylin and eosin staining of colon sections (×40 and ×200, respectively; scale bars, 200 μm and 50 μm, respectively) **(F)**, and the intracellular staining of cytokines in CD4^+^ T cells in the spleens and the colons **(G)**. **(H-K)** Sex- and age-matched *Foxp3*^*Cre*^ and *Foxp1*^*f/f*^*Foxp3*^*Cre*^ mice (*n* = 9–10) were induced with EAE by immunization with MOG peptide and pertussis toxin, and the severity of EAE was evaluated by the clinical score **(H)**, loss of body weight **(I)**, Luxol fast blue-hematoxylin and eosin staining of spinal cord sections (×40, scale bars, 200 μm) **(J)**, and the intracellular staining of cytokines in T cells from the spinal cords and the brains **(K).** Data in **(A-C, H-K)** represent at least three independent experiments. Data in **(D-G)** represent two independent experiments. Data in (**D, H, I**) are mean ± SEM, *P < 0.05 (two-tailed Student *t* test). Data associated with this figure can be found in the supplemental data file **(S1 Data)**. CD, cluster of differentiation; DSS, dextran sulfate sodium; EAE, experimental autoimmune encephalomyelitis; Foxp1, forkhead box P1; IFNγ, interferon gamma; IL, interleukin; MOG, myelin oligodendrocyte glycoprotein; Treg, regulatory T; YFP, yellow fluorescent protein.

We next asked whether Foxp1 plays any role in Treg suppressive function. We first performed phenotype analysis in both 6–8-week-old and >6-month-old mice. Compared with control *Foxp3*^*Cre*^ mice, we found that *Foxp1*^*f/f*^*Foxp3*^*Cre*^ mice had splenomegaly and lymphadenopathy at 8 weeks of age (**S4A Fig**). Flow cytometry analysis showed that the conventional T cells in 8-week-old *Foxp1*^*f/f*^*Foxp3*^*Cre*^ mice exhibited the memory/effector phenotype (CD44^high^CD62L^high^ and CD44^high^CD62L^low^) (**S4B Fig**), with increased percentages of interferon gamma (IFNγ)-producing T cells in the spleens and nonlymphoid organs (**S4C Fig**). *Foxp1*^*f/f*^*Foxp3*^*Cre*^ mice older than 6 months had even higher percentages of CD44^high^CD62L^low^ activated T cells, and more IFNγ or IL-4–producing T cells (**Fig 4A and 4B and S4D Fig**). And strikingly, those older *Foxp1*^*f/f*^*Foxp3*^*Cre*^ mice developed a multi-organ inflammatory disease (**Fig 4C**), strongly suggesting that Foxp1 plays an important role in maintaining the suppressive function of Treg cells under the homeostatic condition.

Treg cells exert an important function in suppressing various inflammatory responses [9]. Thus, we further addressed whether Foxp1 is indispensable for the restriction of inflammatory responses by Treg cells. In the DSS-induced colitis model, we found that with DSS treatment, WT mice survived well and had no weight loss; but in stark contrast, *Foxp1*^*f/f*^*Foxp3*^*Cre*^ mice began to lose weight at d5 post DSS treatment and started to die at d8, with the mortality rate up to 80% at d11 (**Fig 4D and 4E**). Consistent with the severe weight loss and low survival rate, we found that the colons of *Foxp1*^*f/f*^*Foxp3*^*Cre*^ mice treated with DSS exhibited more immune cell infiltration than those from WT mice (**Fig 4F**). We also found that, compared with those from WT mice, the conventional T cells in the spleens and the colons from *Foxp1*^*f/f*^*Foxp3*^*Cre*^ mice had higher percentages of inflammatory cytokines IFNγ- and IL-17A–producing cells (**Fig 4G**). To further confirm these results, we took advantage of the EAE disease model, in which MOG peptide immunization with pertussis toxin (PT) elicits strong T_H_1 and T_H_17 inflammatory responses in vivo [36]. We found that *Foxp1*^*f/f*^*Foxp3*^*Cre*^ mice had obviously higher clinical scores and more body weight loss than did WT mice during the development of EAE disease (**Fig 4H and 4I**). In line with more severe EAE symptoms in *Foxp1*^*f/f*^*Foxp3*^*Cre*^ mice, we observed more demyelination, indicated by the loss of Luxol fast blue staining in spinal cords (**Fig 4J**), more lymphocytes infiltration (**S4E and S4F Fig**), and more IFNγ- or IL-17A–producing T cells in the brains and the spinal cords of *Foxp1*^*f/f*^*Foxp3*^*Cre*^ mice (**Fig 4K**), suggesting that Foxp1-deficient Treg cells have impaired functions in suppressing inflammation in the EAE model. Thus, by using two different inflammatory models, we have demonstrated that Foxp1 is crucial for Treg suppressive function during anti-inflammatory responses.

### Foxp1 regulates iTreg cell differentiation and maintenance

**Fig 5:**
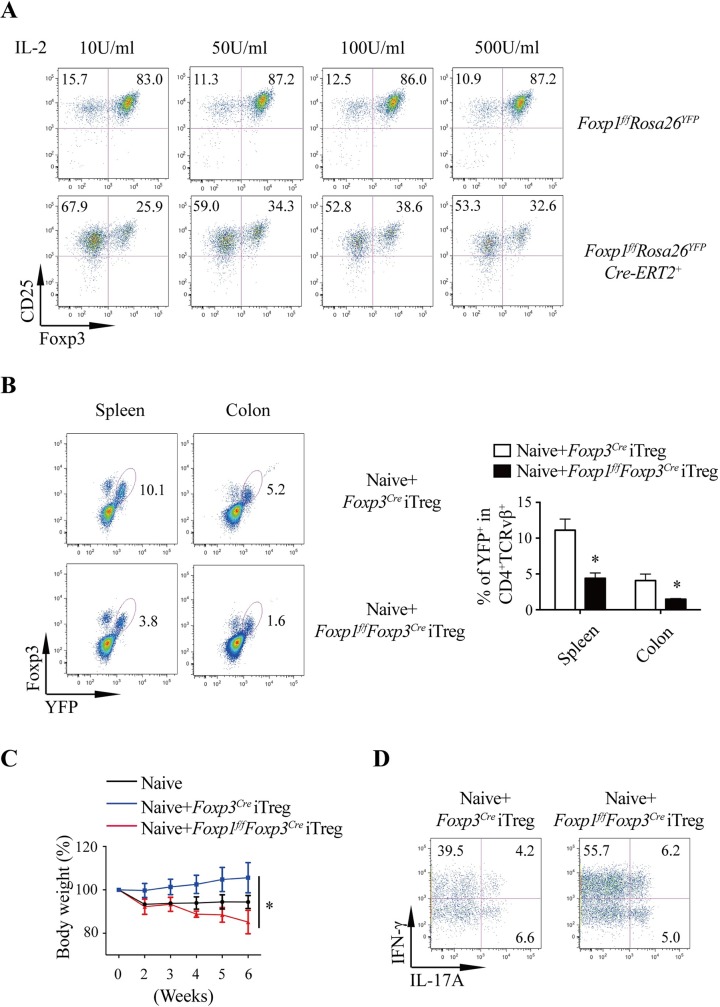
Foxp1 regulates iTreg cell differentiation and maintenance. **(A)** *Foxp1*^*f/f*^*Rosa26*^*YFP*^ and *Foxp1*^*f/f*^Cre-ERT2^+^*Rosa26*^*YFP*^ mice were treated with tamoxifen for 6 days, and CD4^+^ T cells were isolated and cultured in the plate coated with 1 μg/mL anti-CD3/CD28 antibodies in the presence of 0.3 μM 4-hydroxytamoxifen, 5 ng/mL TGF-β, and various concentrations of recombinant human IL-2 for 2 days. Cells were detached and cultured for an additional day. Foxp3 and CD25 expression were analyzed by flow cytometry; numbers adjacent to the outlined areas represent the percentages of gated cells. **(B-D)** Sorted YFP^+^ *Foxp1*^*f/f*^*Foxp3*^*Cre*^ or *Foxp3*^*Cre*^ iTreg cells differentiated in vitro were cotransferred with sorted CD44^low^CD62L^high^CD25^−^ WT naive CD4^+^ T cells into *Rag1*^−*/*−^ mice (*n* = 3). Mice were analyzed at 6 weeks post T-cell transfer. **(B)** Flow cytometry analysis of Foxp3^+^YFP^+^ Treg cells in the spleens and the colons of the recipient mice (left panel) and quantification of YFP^+^ Treg cell frequencies (right panel); the numbers adjacent to the outlined area indicate percentage of YFP^+^ Treg cells. **(C)** The severity of colitis was evaluated by loss of body weight of the recipient mice and **(D)** intracellular staining of cytokines in T cells from the colons; the numbers adjacent to the outlined area indicates percentage of gated cells. Data represent at least two independent experiments. Data in **(B: right panel, C)** are mean ± SEM, *P < 0.05 (two-tailed Student *t* test). Data associated with this figure can be found in the supplemental data file **(S1 Data)**. CD, cluster of differentiation; Foxp1, forkhead box P1; Foxp3, forkhead box P3; IFNγ, interferon gamma; IL, interleukin; iTreg, induced Treg cells; *Rag1*, recombination activating 1; TGF-β, transforming growth factor, beta; Treg, regulatory T; WT, wild-type; YFP, yellow fluorescent protein.

Studies have shown that Foxp3^+^ Treg cells can also be induced from CD4^+^CD25^−^ naive T cells in the periphery or in vitro, which are named pTreg and iTreg cells, respectively [37]. As we have demonstrated that Foxp1 is important for the homeostasis and the suppressive function of tTreg cells (**Fig 2 and Fig 4**), we went further to ask whether Foxp1 plays any role in the differentiation and function of pTreg and iTreg cells. To circumvent the abnormal development of naive CD4^+^ T cells caused by Foxp1 deletion before T cell maturation, we used the inducible Foxp1 conditional knockout mouse line, the *Foxp1*^*f/f*^*Cre-ERT2*^*+*^*Rosa*26^YFP^ mouse [31]. We treated the *Foxp1*^*f/f*^Cre-ERT2^+^*Rosa*26^YFP^ mice with tamoxifen, isolated naive CD4^+^ T cells, and cultured the CD4^+^ T cells under iTreg condition with different concentrations of IL-2. We found that the loss of Foxp1 led to dramatically reduced generation of CD25^high^Foxp3^+^ iTreg cells under all the IL-2 culture conditions (**Fig 5A**), suggesting impaired iTreg differentiation in the absence of Foxp1.

To elucidate the function and maintenance of iTreg cells in vivo, we cotransferred the iTreg cells differentiated in vitro with naive CD4^+^ T cells from the WT mice into the *Rag1*^−*/*−^ recipient mice. Six weeks after cells transfer, we found that the frequency of Foxp1-deficient iTreg cells was significantly lower than that of WT iTreg cells in the spleens and the colons in the recipient mice (**Fig 5B**), suggesting that the maintenance of Foxp1-deficient iTreg cells in vivo is impaired. Consistently, we found that the recipient mice transferred with Foxp1-deficient iTreg cells developed more severe colitis, indicated by the greater body weight loss (**Fig 5C**) and more IFNγ- or IL-17A–producing T cells in the colons (**Fig 5D**). Taken together, our data demonstrated that Foxp1 is important for both iTreg generation and maintenance.

### Foxp1 helps maintain Treg suppressive function through controlling stable Foxp3 expression

**Fig 6:**
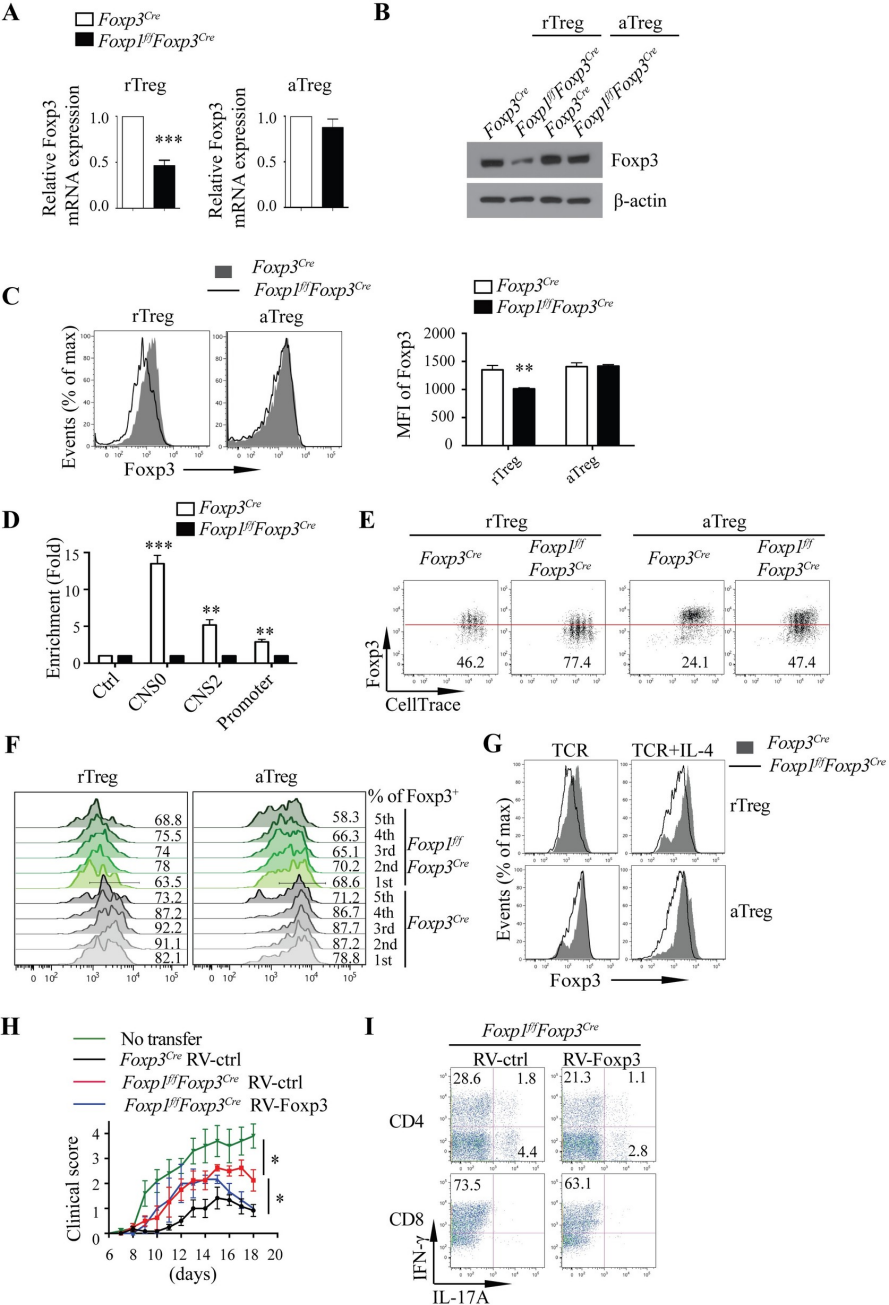
Foxp1 helps maintain Treg cell suppressive function through controlling stable Foxp3 expression. **(A)** Relative Foxp3 mRNA levels in highly purified rTreg (*n* = 4) and aTreg cells (*n* = 6) from *Foxp3*^*Cre*^ and *Foxp1*^*f/f*^*Foxp3*^*Cre*^ mice. **(B)** Immunoblot analysis of Foxp3 in rTreg and aTreg cells from *Foxp3*^*Cre*^ and *Foxp1*^*f/f*^*Foxp3*^*Cre*^ mice. **(C)** Flow cytometry analysis of the expression of Foxp3 in rTreg and aTreg cells from the spleens of *Foxp3*^*Cre*^ and *Foxp1*^*f/f*^*Foxp3*^*Cre*^ mice (left panel), and the corresponding mean fluorescence intensity (MFI) of Foxp3 (right panel) (*n* = 4). **(D)** Foxp1 ChIP was performed in *Foxp3*^*Cre*^ and *Foxp1*^*f/f*^*Foxp3*^*Cre*^ iTreg cells to detect the Foxp1 binding to *Foxp3* CNS0, CNS2, and promoter regions; a region in the *Gmpr* locus was used as a negative control, *n* = 3–4. **(E, F)** Sorted rTreg and aTreg cells from *Foxp3*^*Cre*^ and *Foxp1*^*f/f*^*Foxp3*^*Cre*^ mice were labeled with CellTrace and cultured in the plate coated with 1 μg/mL anti-CD3 and -CD28 antibodies in the presence of 200 U/mL IL-2 for 3 days. **(E)** Foxp3 expression during Treg cell division in rTreg and aTreg cells. The numbers below the red line represent the percentages of Foxp3^low^ Treg cells. **(F)** Foxp3 expression in rTreg and aTreg cells during Treg cell division; numbers adjacent to the histograms represent Foxp3^+^ Treg frequencies. **(G)** Foxp3 expression in *Foxp3*^*Cre*^ and *Foxp1*^*f/f*^*Foxp3*^*Cre*^ rTreg and aTreg cells cultured with plate-bound anti-CD3/CD28 antibodies in the presence or absence of IL-4 for 3 days. **(H-I)** EAE was induced in *Foxp1*^*f/f*^*Foxp3*^*Cre*^ mice by immunization with MOG peptide and pertussis toxin (*n* = 3–8), followed by transfer of sorted *Foxp3*^*Cre*^ and *Foxp1*^*f/f*^*Foxp3*^*Cre*^ Treg cells infected with control retroviruses (RV-ctrl) or retroviruses expressing Foxp3 (RV-Foxp3) at d4. The severity of EAE was evaluated by the clinical score **(H)** and intracellular staining of cytokines in the T cells from the spinal cords and the brains **(I)**; the *Foxp1*^*f/f*^*Foxp3*^*Cre*^ mice without Treg cell transfer were controls. The numbers adjacent to the outlined area indicate the percentage of gated cells. Data in (**A-I**) represent at least three independent experiments. Data in (**A, C**: **right panel, D, H**) are mean ± SEM, *P < 0.05, **P < 0.01, ***P < 0.001 (two-tailed Student *t* test). Data associated with this figure can be found in the supplemental data file **(S1 Data)**. aTreg, activated Treg; CD, cluster of differentiation; ChIP, chromatin immunoprecipitation; CNS0, conserved noncoding sequence 0; CNS2, conserved noncoding sequence 2; d, day; EAE, experimental autoimmune encephalomyelitis; Foxp1, forkhead box P1; Foxp3, forkhead box P3; *Gmpr*, guanosine monophosphate reductase; IL, interleukin; iTreg, induced Treg cells; max, maximum; MFI, mean fluorescence intensity; MOG, myelin oligodendrocyte glycoprotein; rTreg, resting Treg; RV-ctrl, control retrovirus; RV-Foxp3, retrovirus expressing Foxp3; TCR, T-cell receptor; Treg, regulatory T.

To understand the mechanism by which Foxp1 regulates Treg suppressive function, we first examined the expression levels of Foxp3 in Treg cells. We found that the expression of Foxp3 was reduced in Foxp1-deficient rTreg cells at both the mRNA level (**Fig 6A**) and the protein level (**Fig 6B and 6C**), consistent with an earlier observation that the Foxp1-deficient rTreg cells expressed lower levels of YFP, the indicator of Foxp3 expression in mice with a *Foxp3*^*YFP-Cre*^ allele (**S5A Fig**). These results suggest that Foxp1 likely regulates Foxp3 expression in Treg cells at the transcriptional level. Unexpectedly, we found that there was no obvious difference in Foxp3 mRNA and protein levels between WT and Foxp1-deficient aTreg cells (**Fig 6A–6C**), suggesting that somehow the defective Foxp3 expression in the absence of Foxp1 is compensated in the steady-state aTreg cells with a yet unknown mechanism.

To determine whether Foxp1 may directly regulate Foxp3 expression in rTreg cells, we analyzed the CNSs of the Foxp3 locus by bioinformatics. And the conserved forkhead-binding sites were found in the promoter, CNS2, and CNS0 regions of the Foxp3 locus, respectively (**S5B Fig**). Using the chromatin immunoprecipitation (ChIP) approach, we found that Foxp1 bound to these three regions of the Foxp3 locus (**Fig 6D**), suggesting that Foxp3 is a direct target of Foxp1.

It has been reported that CNS2 is required for the maintenance of heritable Foxp3 expression, and it protects Treg lineage identity in inflammatory cytokine environments [15,16]. Because Foxp1 bound to the promoter region and the CNS2 region of Foxp3, we reasoned that Foxp1 might be critical for stable Foxp3 expression, and thereby for Treg stability. Indeed, we found that during cell division upon T-cell receptor (TCR) stimulation, Foxp3 expression was greatly compromised in the Foxp1-deficient rTreg (**Fig 6E** and **6F**). Surprisingly, although the Foxp3 levels were normal in Foxp1-deficient aTreg cells in the steady state (**Fig 6B and 6C**), upon TCR stimulation, the heritable Foxp3 expression was not sustained in Foxp1-deficient aTreg cells (**Fig 6E and 6F**). CNS2 has been shown to help maintain Foxp3 expression under a T_H_2 and T_H_17 cytokine environment [15,16]. We found that the addition of IL-4 to TCR-stimulated Treg cells greatly down-regulated Foxp3 in both Foxp1-deficient rTreg cells and aTreg cells, but not in WT Treg cells (**Fig 6G)**. Similarly, we observed the unstable Foxp3 expression in Foxp1-deficient iTreg cells upon TCR stimulation (**S5C Fig**). Taken together, these results suggest that Foxp1, through its binding to the CNS2 and the promoter regions of the Foxp3 locus, plays a vital role in stabilizing the Foxp3 expression.

Studies have shown that unstable Foxp3 expression affects Treg cell functions [15,16]. We reasoned that the unstable Foxp3 expression in the absence of Foxp1 would contribute to the defective suppressive function of Foxp1-deficient Treg cells. To test this, we constructed a Foxp3 retroviral expression vector (RV-Foxp3) that restored the Foxp3 expression levels in Foxp1-deficient Treg cells to the levels of WT Treg cells (**S5D Fig**). In the EAE model, we found that *Foxp1*^*f/f*^*Foxp3*^*Cre*^ mice, which were transferred with Foxp1-deficient Treg cells overexpressing Foxp3, had significantly remitted symptoms from d15 post EAE induction (**Fig 6H**). Consistently, these mice had fewer IL-17A– and IFNγ-producing T cells in the spinal cords and the brains than *Foxp1*^*f/f*^*Foxp3*^*Cre*^ mice transferred with control Foxp1-deficient Treg cells (**Fig 6I**). All these results suggest that Foxp1-mediated regulation of Foxp3 expression is important for Treg suppressive function.

### Foxp1-dependent transcriptional programs in Treg cells

**Fig 7:**
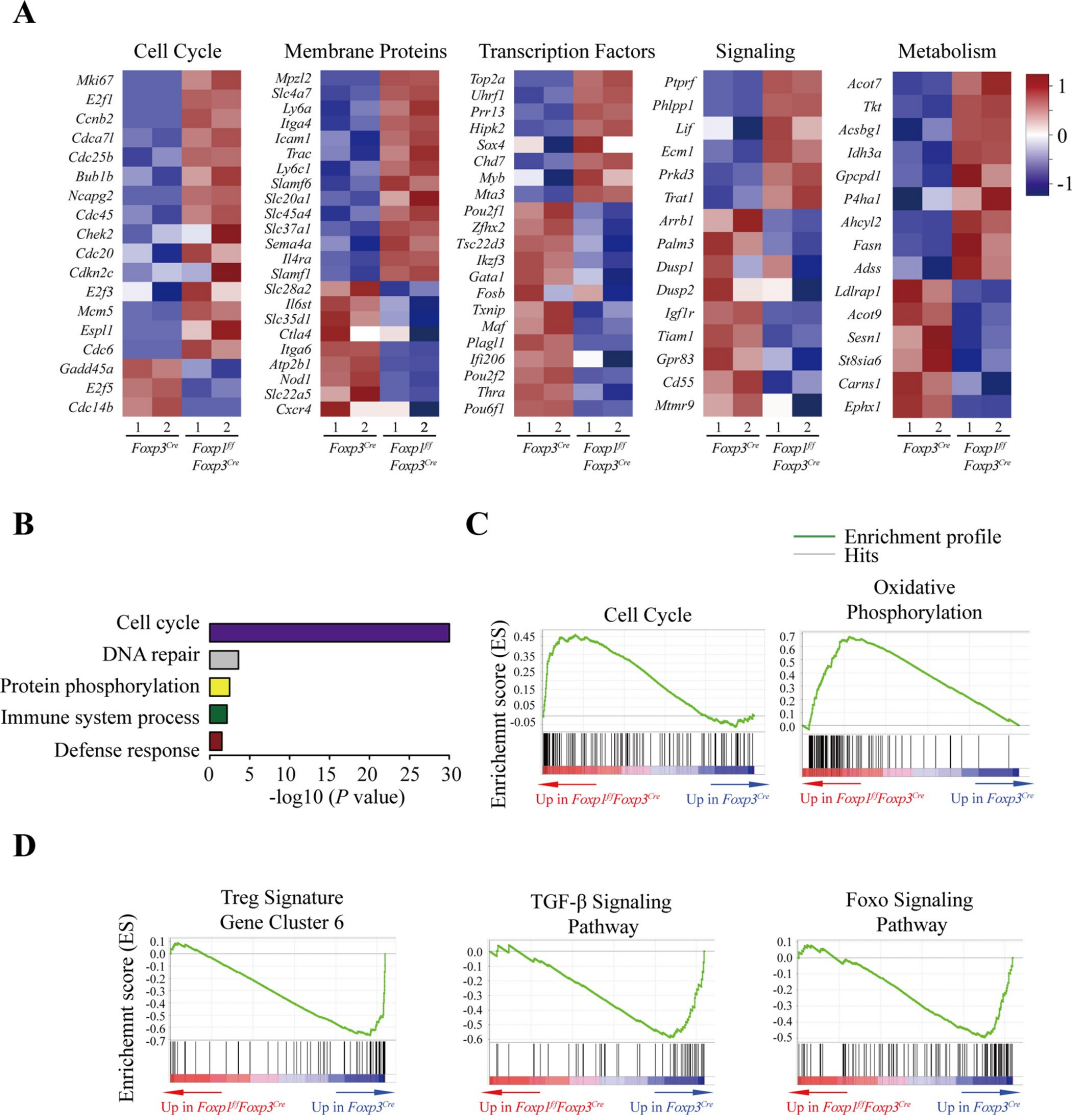
Foxp1-dependent transcriptional programs in Treg cells. **(A)** Heatmap of the representative genes differentially expressed between highly purified *Foxp3*^*Cre*^ and *Foxp1*^*f/f*^*Foxp3*^*Cre*^ rTreg cells (fold change ≥1.5). **(B)** Enriched GO categories of the genes differentially expressed between *Foxp3*^*Cre*^ and *Foxp1*^*f/f*^*Foxp3*^*Cre*^ rTreg cells (Benjamin <0.05). **(C)** Enrichment of gene signatures related to cell cycle and oxidative phosphorylation in *Foxp3*^*Cre*^ versus *Foxp1*^*f/f*^*Foxp3*^*Cre*^ rTreg cells through gene set enrichment analysis (GSEA). Representative enriched gene sets are shown (FDR *q* value <0.05). **(D)** Enrichment of gene signatures related to Treg signature gene cluster 6, TGF-β signaling pathway, and Foxo signaling pathway in *Foxp3*^*Cre*^ versus *Foxp1*^*f/f*^*Foxp3*^*Cre*^ rTreg cells through GSEA. Representative enriched gene sets are shown (FDR *q* value <0.05). Two rounds of RNA sequencing were performed. Data associated with this figure can be found in the supplemental data file **(S1 Data)**. FDR, false discovery rate; Foxo, forkhead box O; Foxp1, forkhead box P1; Foxp3, forkhead box P3; GO, Gene Ontology; GSEA, gene set enrichment analysis; rTreg, resting Treg; TGF-β, transforming growth factor, beta; Treg, regulatory T.

To further gain insight into the mechanism by which Foxp1 regulates Treg cell homeostasis and function, we analyzed the global gene expression profiles of rTreg and aTreg cells from *Foxp1*^*f/f*^*Foxp3*^*Cre*^ and control *Foxp3*^*Cre*^ (WT) mice by RNA sequencing (RNA-Seq). We identified 444 and 400 genes up-regulated or down-regulated, respectively, by more than 1.5-fold in Foxp1-deficient rTreg cells compared with WT rTreg cells, and 218 and 134 genes up-regulated or down-regulated, respectively, by more than 1.5-fold in Foxp1-deficient aTreg cells compared with WT aTreg cells (**S1** and **S2 Tables, Fig 7A** and **S6A Fig**).

In WT aTreg cells, there was a set of up-regulated genes compared with WT rTreg cells (2-fold as threshold). We defined these genes as the putative aTreg cell signature genes. Strikingly, we found that 137 up-regulated genes (31% of total up-regulated genes) in Foxp1-deficient rTreg cells were the aTreg cell signature genes, accounting for approximately 11% of the aTreg signature genes (**S6B** and **S3 Table**), suggesting that Foxp1 is a key transcription factor that normally restrains the differentiation of aTreg cells from rTreg cells.

The differentially expressed genes between WT and Foxp1-deficient rTreg cells were analyzed and found to be clustered into different groups that include cell cycle–regulating genes, transcription factors, genes involved in signaling transduction, and metabolism-related genes (**Fig 7A**), suggesting that Foxp1 regulates many cellular programs in Treg cells. The Gene Ontology (GO) and Kyoto Encyclopedia of Genes and Genomes (KEGG) pathway enrichment analysis showed that the up-regulated genes in Foxp1-deficient rTreg cells were greatly enriched in the cell cycle–related pathway (**Fig 7B**). The gene set enrichment analysis (GSEA) showed similar results, that the up-regulated genes in Foxp1-deficient rTreg cells were significantly enriched in cell cycle and oxidative phosphorylation pathways (**Fig 7C**), suggesting that Foxp1 enforces Treg cell quiescence through regulating cell cycle–and metabolism-related pathways in Treg cells, which is quite similar to our observation in Foxp1-deficient CD8^+^ T cells [38]. To gain more insights into the mechanism by which Foxp1 regulates Treg cell quiescence, we checked the expression of E2 promoter binding factor (*E2f*) family genes and phosphoinositide-3-kinase interacting protein 1 (*Pik3ip1*), which have been shown to regulate CD8^+^ T-cell quiescence [38]. We found that Foxp1 deletion in rTreg cells led to significantly increased expression levels of *E2f1* and its target, cyclin-dependent kinase 1 (*Cdk1*), but decreased expression levels of *Pik3ip1*. However, we did not observe obvious changes in the expression levels of *E2f2* and *E2f3* **(S6C Fig)**. These results suggest that Foxp1 enforces Treg cell quiescence by a similar mechanism involved in regulating CD8^+^ T-cell quiescence.

In contrast to the up-regulated genes associated with Treg cell activation, the down-regulated genes in Foxp1-deficient rTreg cells were greatly enriched in the Treg signature gene cluster 6 [39], the transforming growth factor, beta (TGF-β) signaling pathway, and the Foxo signaling pathway (**Fig 7D**), which are important for Treg identity and function. The Treg signature gene cluster 6 has been shown to contain the signature genes not affected by Foxp3, TGF-β signaling, or TCR activation [39]. Interestingly, our data showed that many genes in this cluster may be regulated by Foxp1 (**Fig 7D**). As TGF-β signaling is important for iTreg differentiation, one of the reasons for the impaired iTreg development of Foxp1-deficient T cells could be the down-regulation of those genes related to the TGF-β signaling pathway in the absence of Foxp1. Collectively, our results suggest that Foxp1 negatively regulates the expression of genes involved in Treg cell activation, and at the same time promotes the expression of a set of genes important for Treg cell identity and function.

### Foxp1 and Foxp3 coordinate the regulation of CTLA-4 expression in Treg cells

**Fig 8:**
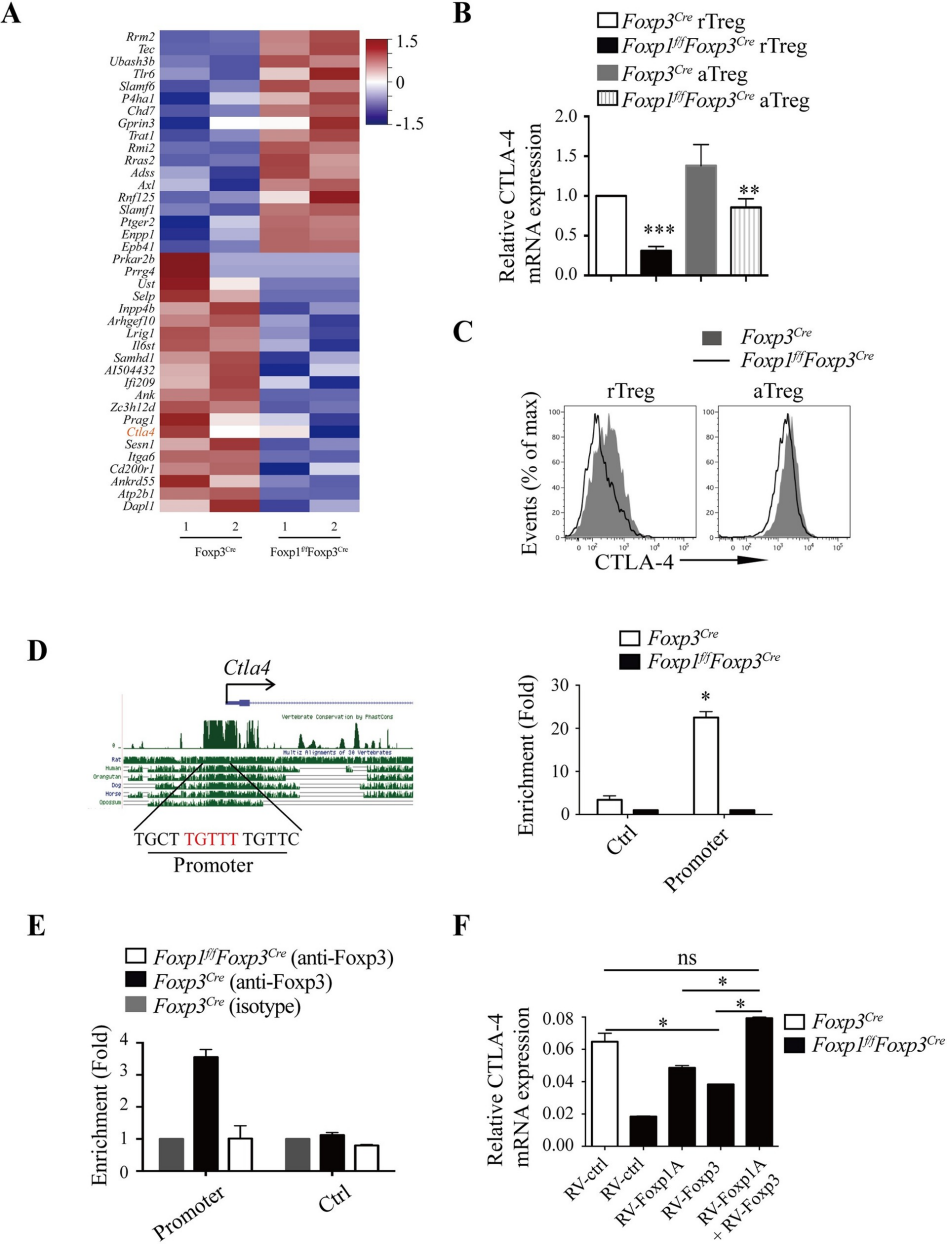
Foxp1 and Foxp3 coordinate the regulation of CTLA-4 expression in Treg cells. **(A)** Heatmap of the representative common target genes of Foxp1 and Foxp3 in rTreg cells. **(B)** The relative mRNA levels of CTLA-4 in purified rTreg and aTreg cells from *Foxp3*^*Cre*^ and *Foxp1*^*f/f*^*Foxp3*^*Cre*^ mice, *n* = 4. **(C)** Flow cytometry analysis of CTLA-4 expression in splenic rTreg and aTreg cells from *Foxp3*^*Cre*^ and *Foxp1*^*f/f*^*Foxp3*^*Cre*^ mice. **(D)** Predicted conserved forkhead-binding site in the *Ctla4* promoter (left panel); Foxp1 ChIP analysis was performed in *Foxp3*^*Cre*^ and *Foxp1*^*f/f*^*Foxp3*^*Cre*^ iTreg cells (right panel) (*n* = 3); −2.4 kb upstream of TSS at the *Ctla4* locus was used as a negative control. **(E)** Foxp3 ChIP was performed in *Foxp3*^*Cre*^ and *Foxp1*^*f/f*^*Foxp3*^*Cre*^ iTreg cells to detect Foxp3 binding to the *Ctla4* promoter region in the presence or absence of Foxp1, *n* = 2. A region in the *Gmpr* locus was used as a negative control. **(F)** The relative mRNA levels of CTLA-4 in *Foxp1*^*f/f*^*Foxp3*^*Cre*^ iTreg cells infected with control retroviruses (RV-ctrl), retroviruses expressing Foxp1A (RV-Foxp1A), retroviruses expressing Foxp3 (RV-Foxp3), or retroviruses expressing both RV-Foxp1A and RV-Foxp3 (RV-Foxp1A+Foxp3), *n* = 3. WT iTreg cells infected with control retroviruses were used as a control. Data in **(A, D-F)** are representative of at least two independent experiments. Data in **(B, C)** are representative of at least three independent experiments. Data in **(B, D, E, F)** are mean ± SEM, *P < 0.05, **P < 0.01, ***P < 0.001 (two-tailed Student *t* test). Data associated with this figure can be found in the supplemental data file **(S1 Data)**. aTreg, activated Treg; ChIP, chromatin immunoprecipitation; CTLA-4, cytotoxic T-lymphocyte-associated protein 4; Foxp1, forkhead box P1; Foxp3, forkhead box P3; *Gmpr*, guanosine monophosphate reductase; iTreg, induced Treg cells; rTreg, resting Treg; Treg, regulatory T; TSS, transcription start site; WT, wild-type.

It has been reported that Foxp subfamily proteins can form homodimers and heterodimers [33,40]. We confirmed the interaction between Foxp1 and Foxp3 in iTreg cells by immunoprecipitation (**S7A Fig**). We reasoned that Foxp1 and Foxp3 might co-regulate some important genes in Treg cells. Studies of Foxp3 ChIP have revealed many Foxp3 target genes in murine Treg cells [41]. By cross-examining the Foxp3 ChIP data set with our RNA-seq data set, we found that about 5% of up- or down-regulated genes in Foxp1-deficient rTreg cells (41 of 844 differential genes) were Foxp3-bound genes, accounting for about 8% of Foxp3 targets (**Fig 8A** and **S4 Table**). These results suggest that Foxp3 and Foxp1 function largely independently in Treg cells.

Nevertheless, CTLA-4, which is critical for Treg suppressive function, was one of the down-regulated genes in Foxp1-deficient Treg cells and among the potential genes co-regulated by Foxp1 and Foxp3 (**Fig 8A** and **S1 Table)**. We recently have shown that Foxp1 directly regulates the expression of CTLA-4 in activated conventional CD4^+^ T cells [42]. Whether CTLA-4 is also a direct target of Foxp1 in Treg cells is not clear. To examine this, we examined the mRNA and protein levels of CTLA-4 in Treg cells. We found that the expression levels of CTLA-4 were decreased in both rTreg and aTreg cells in the absence of Foxp1 (**Fig 8B** and **8C**). Furthermore, Foxp1 ChIP assay in Treg cells showed that Foxp1 bound to the promoter of *Ctla4* (**Fig 8D**), suggesting that CTLA-4 is a direct target of Foxp1 in Treg cells, as well.

It has been reported that Foxp3 can bind to the *Ctla4* promoter. Thus, it is intriguing to ask whether Foxp1 works together with Foxp3 to regulate the expression levels of CTLA-4 in Treg cells. We performed Foxp3 ChIP assay in Foxp1-sufficient and -deficient iTreg cells, and found that the loss of Foxp1 significantly reduced the binding of Foxp3 to the *Ctla4* promoter region (**Fig 8E**). Of note, the protein levels of Foxp3 had only a very minor decrease in Foxp1-deficient iTreg cells compared with those of WT iTreg cells (**S7A Fig**), suggesting that the decreased binding of Foxp3 to *Ctla4* promoter was mainly attributed to the absence of Foxp1. Consistently, although retroviral overexpression of Foxp1 or Foxp3 alone partially rescued the CTLA-4 expression in Foxp1-deficient iTreg cells, overexpression of Foxp1 together with Foxp3 completely rescued the expression of CTLA-4 in Foxp1-deficient iTreg cells. (**Fig 8F and S7B Fig**). Thus, our results demonstrate that Foxp1 and Foxp3 coordinate to regulate the expression levels of CTLA-4.

## Discussion

Foxp1 is an evolutionally conserved transcription factor that is required for the proper development of cardiovascular, intestinal, neural, pulmonary, and lymphoid tissues [29,30,43–47]. In this study, we provided evidence demonstrating that Foxp1 is also an important regulator of Treg cell homeostasis and suppressive function. We found that Foxp1 helps maintain the rTreg cell pool and restrain the differentiation of rTreg cells into aTreg cells. Interestingly, we also found that the ablation of Foxp1 in Treg cells dampens the Treg suppressive function in both homeostatic and pathological situations. Mice with Foxp1 deletion in Treg cells spontaneously developed inflammatory disease, and DSS-induced colitis and EAE were more severe in *Foxp1*^*f/f*^*Foxp3*^*Cre*^ mice. Mechanistically, we found that Foxp1 stabilizes Foxp3 expression to maintain both the Treg suppressive function in anti-inflammatory responses and the Treg cell stability under TCR stimulation or destabilizing conditions. In addition, we found that CTLA-4 is a direct target of Foxp1 in Treg cells, and the absence of Foxp1 attenuates the Foxp3 binding to the *Ctla4* promoter. Our study identifies Foxp1 as a critical regulator of Treg cell quiescence, suppressive function, and stability in homeostatic and inflammatory responses.

Akin to conventional CD4^+^ T cells, tTreg cells can be divided into resting and activated subpopulations with relatively different functions. While rTreg cells that reside in lymphoid tissues play crucial roles in preventing lymphoproliferative disease, aTreg cells are the dominant Treg cell population in nonlymphoid tissues as potent suppressors in anti-inflammatory responses [4,5]. The balance between rTreg and aTreg is important for immune homeostasis. In our study, we found that Foxp1 deficiency leads to a gradual loss of rTreg cells. Given that rTreg cells function as suppressors of lymphoproliferation, the reduced rTreg cell number may explain the early splenomegaly and lymphadenopathy in young Foxp1-deficient mice. Unexpectedly, despite the increased numbers of aTreg cells, *Foxp1*^*f/f*^*Foxp3*^*Cre*^ mice gradually develop spontaneous inflammatory diseases with age, and have more severe disease in DSS-induced colitis and EAE models, indicating that, although Foxp1 helps restrain the differentiation of aTreg cells from rTreg cells, it is also important for the suppressive function of aTreg cells.

In addition to tTreg cells, studies have shown that Foxp3^+^ Treg cells can also develop in the periphery from mature conventional CD4^+^ T cells (iTreg cells) [37]. It is an intriguing question whether Foxp1 is important for both tTreg cells and iTreg cells. Recently, Ghosh and colleagues showed that Foxp1 is vital for the iTreg cell differentiation and functions but is dispensable for the function of tTreg cells [48]. However, by using the same *Foxp1*^*f/f*^ mouse line, Konopacki and colleagues showed that Foxp1 has an essential function in regulating tTreg cells [49]. The fact that Ghosh and colleagues did not observe functional defects in Foxp1-deficient tTreg cells could be due to some Foxp1^+^ Treg cells present in their *Foxp1*^*f/f*^*Foxp3*^*Cre*^ mice [48]. In our study, we have used a different *Foxp1*^*f/f*^ mouse line: Foxp1 is efficiently deleted in Treg cells in our *Foxp1*^*f/f*^*Foxp3*^*Cre*^ mice (**Fig 1A**) [30,31], and we demonstrated that Foxp1 is important for the differentiation and suppressive function of tTreg cells. Thus, we conclude that Foxp1 plays vital roles in both tTreg and iTreg cells.

In conventional naive T cells, Foxp1 actively maintains T-cell quiescence largely by restraining IL-7Rα expression [31]. However, in this study we did not find that Foxp1 regulates the expression of IL-7Rα in rTreg cells, suggesting that the mechanisms underlying the transcriptional regulation of IL-7Rα are different between conventional CD4^+^ T and Treg cells. Our earlier study has also shown that Foxp1 does not control IL-7Rα expression in pre-B cells that express high levels of Foxp1 and IL-7Rα [29]. Thus, for the same target gene but in different types of cells, Foxp1 seems to engage different transcriptional partners or complexes to exert its regulation. Although Foxp1 does not control IL-7Rα expression in Treg cells, we found that Foxp1 regulates the expression levels of *E2f1* and *Pik3ip1* in Treg cells, two genes involved in the regulation of CD8^+^ T cell quiescence [38], suggesting that Foxp1 enforces Treg quiescence in a similar way as in CD8^+^ T cells: by simultaneously repressing key pathways in cellular metabolism and cell cycle progression.

The work of Ghosh and colleagues shows that Foxp1 binds to the promoter and the CNS2 region of the Foxp3 locus, which retains permissive histone modifications [48]. In our study, we have confirmed that Foxp1 binds to the promoter and the CNS2 region of the Foxp3 locus and, additionally, the CNS0 region. We find that Foxp1 sustains de novo expression of Foxp3 in rTreg cells and maintains stable Foxp3 expression in both rTreg and aTreg cells upon TCR stimulation. Previous studies have reported that Foxp3 CNS2 functions as a sensor of TCR signal to regulate and maintain Foxp3 expression [15,16]. Thus, it is possible that in quiescent rTreg cells, the control of Foxp3 expression by Foxp1 is mainly via promoter or CNS0, but may not involve CNS2; however, upon TCR stimulation, Foxp1 may engage in the formation of the loop between the promoter and the CNS2 of *Foxp3* locus, which is required for the maintenance of high levels of Foxp3 expression. Several proteins such as Gata3, Ets-1, CREB, Stat5, Foxo1, Foxp3, Runx1, and CBFβ have been shown to bind to the promoter or the CNS2 region of *Foxp3* gene [17–21,23,24,27,50]. This raises interesting questions about whether Foxp1 interacts with those proteins, especially, with Foxp3 in the regulation of Foxp3 expression and whether Foxp1 plays a nonredundant role in the regulation of Foxp3 expression. Others’ and our data show that Foxp1 and Foxp3 can form heterodimers. Thus, it is interesting to propose that the formation of the regulatory loop between the promoter and the CNS2 of the *Foxp3* locus requires Foxp1 and Foxp3 heterodimers, which warrants further investigation.

A variety of molecules are involved in the suppressive function of Treg cells, including CTLA-4, a key “checkpoint” in immune tolerance. CTLA-4 deficiency in Treg cells impairs their suppressive function both in vivo and in vitro [51]. CTLA-4 is also known as a Foxp3 direct target. In our study, we showed that Foxp1 and Foxp3 coordinate the regulation of CTLA-4 expression levels. In the absence of Foxp1, Foxp3 binds less to the promoter of *Ctla4*, suggesting that Foxp3-mediated CTLA-4 regulation is dependent on the presence of Foxp1. As Foxp1 can form heterodimers with Foxp3, it is also interesting to ask how many Foxp3 targets are regulated by Foxp1/Foxp3 dimers. In our RNA-Seq data, although the potential common targets of Foxp1 and Foxp3 are not too many, these genes, such as CTLA-4 and Foxp3, seem to be important for Treg function and identity. Thus, how Foxp1 homodimers or heterodimers with other proteins contribute to Treg cell differentiation and Treg suppressive function is worth further investigation.

In summary, we have defined the critical roles of another Foxp subfamily transcription factor, Foxp1, in regulating Treg cell homeostasis and function. In addition, the interplay between Foxp1 and Foxp3 in regulating key Treg genes also provides new understanding of the transcriptional programs in Treg cells.

## Materials and methods

### Ethics statement

All procedures were carried out in accordance with guidelines of the Animal Facility of IPS. The experimental procedures were approved by the Institutional Animal Care and Use Committee of Institut Pasteur of Shanghai, China (A2019014). All efforts were made to minimize suffering.

### Mice

*Foxp1*^*f/f*^, *Cre-ERT2*^*+*^, and *Rosa*^*YFP*^ transgenic mice have been previously described [30,31]. *Foxp1*^*f/f*^ were bred with *Foxp3*-YFP-*Cre* knock-in mice from Jackson Laboratories to generate *Foxp1*^*f/f*^*Foxp3*^*Cre*^ mice. Control animals were age-/sex-matched control *Foxp3*^*Cre*^ mice (WT) or *Foxp1*^*f/f*^ mice. Unless otherwise stated, all experiments were performed with mice at 6–8 weeks of age. All mice were maintained under specific pathogen-free conditions at Institute Pasteur of Shanghai, China.

### Flow cytometry, cell sorting, and intracellular staining

These procedures were done as described previously [31]. The sorted cell populations were >98% pure. Antibodies used in FACS analysis and cell sorting were as follows: APC-Cy7-anti-CD4 (GK1.5, Thermo Fisher Scientific, Waltham, MA), PE-Cy7-anti-CD4 (GK1.5, Thermo Fisher Scientific), PE-Cy7-anti-CD8 (53–6.7, Thermo Fisher Scientific), PerCP-Cy5.5-anti-CD8 (53–6.7, BioLegend, San Diego, CA), PE-anti-CD44 (IM7, Thermo Fisher Scientific), APC-anti-CD62L (MEL-14, Thermo Fisher Scientific), PE-Cy7-anti-CD25 (PC61.5, Thermo Fisher Scientific), Pacific Blue-anti-Foxp3 (FJK-16s, Thermo Fisher Scientific), APC-anti-Foxp3 (FJK-16s, Thermo Fisher Scientific), PerCP-Cy5.5-anti-interferon-γ (XMG1.2, Thermo Fisher Scientific), Alexa Fluor 700-anti-IL-17A (TC11-18H10, BD Pharmingen), Pacific Blue-anti-Granzyme B (NGZB, Thermo Fisher Scientific), PE-anti-CTLA-4 (UC10-4B9, BioLegend), BV605-anti-CD44 (IM7, BioLegend), and Alexa Fluor 647-goat-anti-rabbit IgG (A-21244, Thermo Fisher Scientific). Dead cells were excluded through live/dead staining by using Live/Dead Fixable Aqua Dead Cell staining kit (Thermo Fisher Scientific).

For intracellular staining of Foxp1, the staining procedures were performed as described previously [32]. For intracellular staining of Foxp3 and CTLA-4, cells were fixed with 3.7% formaldehyde following cell surface staining (antibodies mentioned above) and were permeabilized with 0.2% Triton X-100. Then, cells were stained with Pacific Blue-anti-Foxp3 or PE-anti-CTLA-4 at 4°C for 90 minutes. For APC-anti-Foxp3 staining, we used the Foxp3 staining kit (Thermo Fisher Scientific) and performed it as per the manufacturer’s instructions.

For intracellular staining of cytokines, cells were stimulated for 6 hours with 10 ng/mL PMA (phorbol 12-myristate 13-acetate, Sigma, St. Louis, MO) and 1 μg/mL ionomycin (Sigma). Before staining, cells were treated for 2 hours with 10 μg/mL Brefeldin A (BFA; Sigma). The intracellular staining was done as described [31].

### iTreg cell differentiation and transfer

*Foxp1*^*f/f*^*Rosa26*^*YFP*^ and *Foxp1*^*f/f*^ *Cre-ERT2*^*+*^*Rosa26*^*YFP*^ mice were treated daily with tamoxifen (Sigma) (2 mg per mouse) for 5 days and rested for 1 day. CD4^+^ T cells were isolated and cultured with 1 μg/mL plate-bound anti-CD3 (BioXcell) and anti-CD28 (BioXcell), 5 ng/mL TGF-β, and indicated recombinant human IL-2 in the presence of 0.3 μM 4-hydroxytamoxifen (Sigma) for 2 days, followed by detaching and culturing in T-cell medium (Dulbecco’s Modified Eagle Medium [DMEM] containing 10% heat-inactivated FCS, 2 mM L-glutamine, penicillin-streptomycin, nonessential amino acids, sodium pyruvate, vitamins, 10 mM HEPES and 50 μM 2-mercaptoethanol) in the presence of 5 ng/mL TGF-β and the indicated recombinant human IL-2.

For iTreg cell transfer, naive (YFP^–^CD4^+^CD44^low^CD62L^high^) T cells from the spleens and the lymph nodes of *Foxp3*^*Cre*^ and *Foxp1*^*f/f*^*Foxp3*^*Cre*^ mice were sorted by FACS Aria II device (BD Bioscience, San Jose, CA). Naive T cells were stimulated for 48 hours with plate-bound anti-CD3 (5 μg/mL, 145-2C11, BioXcell) and anti-CD28 (5 μg/mL, 37.51, BioXcell) in complete T-cell medium supplemented with TGF-β (5 ng/mL, R&D Systems, Minneapolis, MN) and IL-2 (200 U/mL, Beijing Four Rings Biopharmaceutical Co., Beijing, China). Then, the cells were detached and cultured in T-cell medium containing 5 ng/mL TGF-β and 200 U/mL recombinant human IL-2 for an additional 3 days. Afterwards, 4 × 10^5^ sorted YFP^+^ iTreg cells were cotransferred with 1 × 10^6^ purified WT naive CD4^+^ T cells (CD4^+^CD44^low^CD62L^high^CD25^–^) into *Rag1*^−*/*−^ mice to perform the colitis assay.

### Quantitative RT-PCR

rTreg and aTreg cells from *Foxp3*^*Cre*^ or *Foxp1*^*f/f*^*Foxp3*^*Cre*^ mice were sorted out by a FACS Aria II device (BD Bioscience). Then, total RNA was isolated from whole cells using the TRIZOL reagent (Life technologies, Carlsbad, CA) according to the manufacturer’s instructions. Complementary DNA was reverse transcribed using the PrimeScript RT reagent kit (Takara, Tokyo, Japan). Quantitative PCR was performed with SYBR Green mix (Takara) on ABI Prism 7500 sequence detection system (Applied Biosystems). Relative mRNA expression of genes was normalized to GAPDH. The primers are listed as follows: *Foxp1*, forward: CTGAATCTGGTATCAAGTGTCACCCTCT, reverse: GATTCGAGAATGGCCTGCCTGA; *Foxp3*, forward: CAGAGTTCTTCCACAACATGGAC, reverse: CAGGGATTGGAGCACTTGTTG; *Ctla4*, forward: AGAACCATGCCCGGATTCTG, reverse: GCTCTGTTGGGGGCATTTTC; *GAPDH*, forward: TTCACCACCATGGAGAAGGC, reverse: GGCATGGACTGTGGTCATGA; *E2f1*, forward: GCATCCAGCTCATTGCCAAGAAGT, reverse: GAAAGCAGTTGCAGCTGTGTGGTA; *E2f2*, forward: TCAGAGTTGCTCCCTGAGCTTCAA, reverse: TTGAAGTTGCCTACGGCACGGATA; *E2f3*, forward: AGGGCCCATTGAGGTTTACTT, reverse: GTAGAAACCGAGCAGTCACTA; *Cdk1*, forward: CATGGACCTCAAGAAGTACCTGG, reverse: CAAGTCTCTGTGAAGAACTCGCC; *Mcm5*, forward: GGAGGCATTGAGACTGTTCCAG, reverse: AGACACCTGAGAGCCAATGGCA; and *Pik3ip1*, forward: ATCCTCGCTATTGGAGCTGGCATT, reverse: TCTGGTTGCTGTGCACAATGATGG.

### Immunoblot and immunoprecipitation

Cells were washed with ice-cold PBS twice and lysed in RIPA buffer (20 mM Tris-HCl, pH 7.5; 150 mM NaCl; 1% NP-40; 0.1% SDS; 0.5% sodium deoxycholate) containing 1× Protease Inhibitor Cocktail (Roche, Mannheim, Germany). Cell extracts were separated by SDS-PAGE and transferred to a polyvinylidene difluoride membrane (Bio-Rad, Hercules, CA). The membrane was analyzed by immunoblot with rabbit anti-Foxp1 [31], mouse anti-Foxp3 (eBio7979, Thermo Fisher Scientific), or mouse anti-β-actin (AC74, Sigma). For co-immunoprecipitation, cell lysates were immunoprecipitated with appropriate antibodies (1 μg/mL) using protein A sepharose beads (GE Healthcare, Uppsala, Sweden) at 4 °C. Samples were used for immunoblot analysis with the indicated antibodies.

### Treg cell proliferation

YFP^+^ CD44^low^CD62L^high^CD4^+^ T cells (rTregs) and YFP^+^ CD44^high^CD62L^low^CD4^+^ T cells (aTregs) sorted from *Foxp3*^*Cre*^ or *Foxp1*^*f/f*^*Foxp3*^*Cre*^ mice were labeled with CellTrace following the manufacturer’s instruction (CellTrace Violet Cell Proliferation Kit, Thermo Fisher Scientific). To assess the proliferation of purified Treg cells to TCR stimulation, labeled Treg cells were stimulated with the indicated concentration of anti-CD3 and anti-CD28 in the presence of 200 U/mL IL-2 for 3 days.

### Retroviral transduction

The open reading frames of Foxp3 and Foxp1A were subcloned into the retroviral vector MSCV-IRES-VEX and MSCV-IRES-Tomato. Retroviruses were packaged in the HEK293T cell line as described previously [32]. Briefly, retroviral expression plasmids were transfected into HEK293T cells using Lipofectamine 2000 (Thermo Fisher Scientific). For transduction of retrovirus, purified Treg cells were stimulated with 10 μg/mL anti-CD3 and 10 μg/mL anti-CD28 in the presence of 500 U/mL IL-2 for 24 hours. Activated cells were spin transduced with 1 mL virus-containing medium with polybrene (6 μg/mL) at 650*g*, 30°C for 2 hours; transduced cells were cultured with complete T-cell medium supplemented with recombinant human IL-2 (500 U/mL). The cells were harvested on d4 for staining or adoptive transfer.

### Colitis induction and lamina propria mononuclear cell isolation

For the induction of acute colitis, age-matched female *Foxp3*^*Cre/Cre*^ or *Foxp1*^*f/f*^*Foxp3*^*Cre/Cre*^ mice were treated with 2.5% DSS (molecular weight 36,000–50,000 kDa; MP Biomedicals, Aurora, OH) dissolved in drinking water for 14 days. Mice were weighed every day and killed at d14. Cytokine production of CD4^+^ T cells from spleens and colons were analyzed by flow cytometry. The colons were also processed for tissue sectioning and HE staining.

For isolation of lamina propria (LP) mononuclear cells, dissected colons were washed with PBS followed by removing fat tissues and Peyer’s patches. Colon tissues were pretreated in 5% FBS DMEM containing 1 mM DTT (Sigma) and 30 mM EDTA (AMResco) at 37°C for 30 minutes in a shaker. Then, pretreated tissues were digested in 5% FBS DMEM containing Liberase (25 μg/mL, Roche) and DNase I (20 μg/mL, Sigma) at 37°C for 30 minutes in a shaker. Digested tissues were meshed in a 70-μm cell strainer, followed by percoll gradient (80%/40%) centrifugation. Mononuclear cells present in the interphase were collected, washed with PBS, and resuspended in T-cell medium.

### EAE induction, Treg administration, and CNS mononuclear cell isolation

For EAE induction, 2-month-old *Foxp3*^*Cre*^ or *Foxp1*^*f/f*^*Foxp3*^*Cre*^ mice were immunized subcutaneously with 200 μg of MOG35–55 peptide (GL Biochem, Shanghai, China) emulsified in Complete Freund’s Adjuvant (CFA; Sigma). Additionally, mice were injected intravenously with 200 ng of PT (List biological laboratories, Campbell, CA) on d0 and d2 following immunization. For Treg cell transfer, experimental mice were intravenously injected with 2 × 10^6^ Foxp3-transduced *Foxp1*^*f/f*^*Foxp3*^*Cre*^ Treg cells in 200 μL PBS at d4 post EAE induction. Control groups were either injected with the same number of *Foxp3*^*Cre*^ or *Foxp1*^*f/f*^*Foxp3*^*Cre*^ Treg cells infected with control retroviruses (RV-ctrl) or retroviruses expressing Foxp3 (RV-Foxp3) at d4 post EAE induction. The clinical scores and body weight of mice was evaluated daily after 6 days following immunization. Severity of EAE was assessed daily with a 0–5 scoring system [52]: 0, no clinical signs; 0.5, partially limp tail; 1, paralyzed tail; 2, loss in coordinated movement, hind limb paresis; 2.5, one hind limb paralyzed; 3, bilateral hind limb paralysis; 3.5 hind limbs paralyzed, weakness in forelimbs; 4, forelimb paralysis; 5, moribund.

For isolation of CNS mononuclear cells, mice were perfused with PBS to remove blood cells within tissues. CNS tissues (brains and spinal cords) were cut into small pieces and digested with Liberase (25 μg/mL, Roche) and DNaseI (20 μg/mL, Sigma) at 37°C for 30 minutes. Digested tissues were meshed in a 70-μm cell strainer, followed by percoll gradient (80%/40%) centrifugation. Mononuclear cells present in the interphase were collected, washed by PBS, and resuspended in culture medium.

### Histopathology

For histological analysis, mouse tissues were fixed in 3.7% (vol/vol) formaldehyde, and sections were stained with hematoxylin and eosin, or Luxol fast blue-hematoxylin and eosin according to standard procedures.

### ChIP

Naive CD4^+^ T cells from *Foxp3*^*Cre*^ or *Foxp1*^*f/f*^*Foxp3*^*Cre*^ mice were cultured under iTreg-polarizing conditions (5 ng/mL TGF-β, 200 U/mL IL-2). ChIP of Foxp1, Foxp3 was performed as described [32]. Immunoprecipitated DNA and input DNA were analyzed by quantitative PCR (Applied Biosystems). The primers were as follows: *Foxp3* CNS2, forward: ATCTGGCCAAGTTCAGGTTG, reverse: GGCGTTCCTGTTTGACTGTT; *Foxp3* promoter, forward: CCCTGCAATTATCAGCACAC, reverse: TGTGGGAAACTGCCACATTA; *Ctla4* promoter region, forward: GACTCCACGTCTCCAGGTCCTCAG, reverse: GGAAGCCGTGGGTTTAGCTGTTAC; *Gmpr*, forward: CAGCTGGAACAGCCTTGGAA, reverse: AAATGTCAAGGCCCCTGTGA. Control for CTLA4 promoter region in Foxp1 ChIP assay: forward: GTCAGAGTATTTTATCACAGCCAC, reverse: AGTCTACTGCAAAACCCCAAG.

### RNA-Seq analysis

#### RNA sequencing

Total RNA was extracted using the TRIZOL reagent (Life technologies) according to the manufacturer’s instructions. RNA quality was assessed and sequenced using illumina Hiseq X platform in Novogene (Beijing, China).

#### RNAseq data analysis

Raw data from the sequencer first underwent quality control using FastQC. Next, reads were treated using trim_galore, in which illumina adapter sequence and low-quality reads(phred score <20) at the 3′ end were trimmed, and the paired reads were removed if any of the two reads did not meet the minimum length (20 bp). After that, duplicates were removed using Picard. For each sample, we counted the reads hits on each transcript using htseq-count.

#### Differential analysis

Differential analysis between *Foxp3*^*Cre*^ or *Foxp1*^*f/f*^*Foxp3*^*Cre*^ Treg cell groups was done by a count-based method, limma, which is implemented in R, and voom is involved for normalization [53,54]. Paired samples analysis was employed to compensate batch effect. Significantly expressed genes were first screened by BH-adjusted *P* value 0.05 and further filtered with 1.5 fold-change and 2 fold-change for *Foxp1*^*f/f*^*Foxp3*^*Cre*^ Treg cells versus *Foxp3*^*Cre*^ Treg cells, and WT aTreg cells versus WT rTreg cells, respectively.

#### Functional analysis

DAVID

Significant genes identified through comparisons between *Foxp1*^*f/f*^*Foxp3*^*Cre*^/*Foxp3*^*Cre*^ or aTreg/rTreg were further used for enrichment analysis using DAVID. Biological processes (BPs) in GO and pathways in KEGG were chosen as significantly enriched terms with a Benjamini value less than 0.05.

GSEA

In parallel, we used GSEA v3.0 (Broad Institute, PreRanked mode) for enrichment analysis. To be consistent with the way for identifying significant genes, we used the *t* statistic output from limma as the metrics for ranking. A total of 1,000 gene set permutations were set as default, and gene sets were obtained through collecting pathways from KEGG and Biocarta, as well as BPs from GO. A gene set with an FDR *q* value less than 0.05 was considered significantly enriched.

Heatmap

For the heatmap, the log2 scale of the fragments per kilobase of transcripts per million fragments mapped (FPKM) values of the significant genes were used as input for heatmap generation.

### Statistics

Statistical analyses were performed with Prism 6.0 (GraphPad). Two-tailed Student *t* tests and paired *t* tests were used for the calculation of *P* values, except for those in RNA-Seq analysis. *P < 0.05, **P < 0.01, and ***P < 0.001.

## Supporting information

S1 Fig The gating strategy for flow cytometry analysis and cell sorting. The gating strategy of CD44^low^CD62L^high^ rTreg and CD44^high^CD62L^low^ aTreg (upper panel), and the purity check after cell sorting of CD44^low^CD62L^high^ rTreg and CD44^high^CD62L^low^ aTreg in *Foxp3*^*Cre*^ and *Foxp1*^*f/f*^*Foxp3*^*Cre*^ mice (lower panel). aTreg, activated Treg; rTreg, resting Treg.
(TIF)

S2 Fig The expression of Foxp1 in Treg cells from different tissues. Intracellular staining of Foxp1 in Treg cells in spleens, lungs, livers, small intestines (SI) from *Foxp3*^*Cre*^ mice (WT) (upper panel), and the corresponding MFI of Foxp1 (lower panel). Foxp1-deficient Treg cells were used as staining controls. Data are representative of two independent experiments. Data associated with this figure can be found in the supplemental data file **(S1 Data)**. Foxp1, forkhead box P1; MFI, mean fluorescence intensity; SI, small intestine; Treg, regulatory T; WT, wild-type.
(TIF)

S3 Fig Cell proliferation and apoptosis in *Foxp3^Cre^* and *Foxp1^f/f^Foxp3^Cre^* Treg cells. **(A)** Flow cytometry analysis of Ki-67 expression in rTreg and aTreg cells in spleens from 3-week-old *Foxp3*^*Cre*^ and *Foxp1*^*f/f*^*Foxp3*^*Cre*^ mice; numbers above bracketed lines indicate percent Ki-67^+^ Treg cells. **(B)** Proliferation of aTreg cells cultured with different concentrations of anti-CD3 and anti-CD28 antibodies in the presence of 200 U/mL IL-2 for 3 days, as shown by dilution of CellTrace; numbers above bracketed lines indicate percent proliferating cells. **(C)** Annexin V and 7-AAD staining in rTreg and aTreg cells in spleens from 6-week-old *Foxp3*^*Cre*^ and *Foxp1*^*f/f*^*Foxp3*^*Cre*^ mice; numbers adjacent to the outlined area represent the percentage of gated cells. Data are representative of two independent experiments. aTreg, activated Treg; IL, interleukin; Ki-67, antigen identified by monoclonal antibody Ki 67; rTreg, resting Treg.
(TIF)

S4 Fig Lymphadenopathy and activated T-cell phenotype in *Foxp1^f/f^Foxp3^Cre^* mice. **(A)** Representative picture of spleens and lymph nodes from 8-week-old sex- and age-matched *Foxp3*^*Cre*^ and *Foxp1*^*f/f*^*Foxp3*^*Cre*^ mice. **(B)** Flow cytometry analysis of CD44 and CD62L expression in conventional CD4^+^ T cells (YFP^−^CD4^+^) and CD8^+^ T cells in spleens and lymph nodes from 8-week-old *Foxp3*^*Cre*^ and *Foxp1*^*f/f*^*Foxp3*^*Cre*^ mice; numbers adjacent to the outlined area represent the percentage of gated cells. **(C)** Intracellular staining of cytokines in conventional CD4^+^ and CD8^+^ T cells in spleens, livers, and small intestines (SI) from 8-week-old *Foxp3*^*Cre*^ and *Foxp1*^*f/f*^*Foxp3*^*Cre*^ mice; numbers adjacent to the outlined area represent the percentage of gated cells. **(D)** Intracellular staining of IL-4 in YFP^−^CD4^+^ T cells in the spleens from 28-week-old *Foxp3*^*Cre*^ and *Foxp1*^*f/f*^*Foxp3*^*Cre*^ mice (left panel) and the corresponding statistics (right panel) (*n* = 3). Numbers adjacent to the outlined area indicate the percentage of IL-4^+^ cells in CD4^+^ T cells. **(E)** Hematoxylin and eosin staining of spinal cord sections (×100, scale bars, 100 μm) from *Foxp3*^*Cre*^ and *Foxp1*^*f/f*^*Foxp3*^*Cre*^ mice induced with EAE by immunization with MOG peptide and PT. **(F)** Quantification of CD4^+^ and CD8^+^ T-cell frequencies in alive cells from brains and spinal cords of *Foxp3*^*Cre*^ or *Foxp1*^*f/f*^*Foxp3*^*Cre*^ mice (*n* = 12–17). Data in (**A-C**, **F**) are representative of at least three independent experiments. Data in (**D** and **E**) are representative of two independent experiments. Data in **(D: right panel, F)** are mean ± SEM, **P < 0.01, ***P < 0.001 (two-tailed Student *t* test). Data associated with this figure can be found in the supplemental data file **(S1 Data)**. CD, cluster of differentiation; EAE, experimental autoimmune encephalomyelitis; IL, interleukin; MOG, myelin oligodendrocyte glycoprotein; PT, pertussis toxin; SI, small intestine.
(TIF)

S5 Fig Foxp1 sustains stable expression of Foxp3. **(A)** Flow cytometry analysis of YFP expression in rTreg and aTreg cells from the spleens of *Foxp3*^*Cre*^ and *Foxp1*^*f/f*^*Foxp3*^*Cre*^ mice. **(B)** Predicted conserved forkhead-binding sites (highlighted in red) in the CNS0, promoter, and CNS2 region of *Foxp3*, respectively. **(C)** Sorted YFP^+^ *Foxp3*^*Cre*^ and *Foxp1*^*f/f*^*Foxp3*^*Cre*^ iTreg cells were stimulated with anti-CD3 and -CD28 beads for 3 days, and intracellular staining of Foxp3 in *Foxp3*^*Cre*^ and *Foxp1*^*f/f*^*Foxp3*^*Cre*^ iTreg cells were analyzed by flow cytometry. (**D)** Intracellular staining of Foxp3 in *Foxp3*^*Cre*^ and *Foxp1*^*f/f*^*Foxp3*^*Cre*^ iTreg cells infected with control retroviruses (RV-ctrl) or retroviruses expressing Foxp3 (RV-Foxp3) (left panel), and the corresponding MFI of Foxp3 (right panel). Data in (**A, C**) are representative of at least three independent experiments. Data in (**D**) are representative of two independent experiments. Data associated with this figure can be found in the supplemental data file **(S1 Data)**. aTreg, activated Treg; CNS, conserved noncoding sequence; Foxp1, forkhead box P1; Foxp3, forkhead box P3; iTreg, induced Treg cells; MFI, mean fluorescence intensity; rTreg, resting Treg; YFP, yellow fluorescent protein.
(TIF)

S6 Fig Transcriptional programs regulated by Foxp1. **(A)** Representative genes differentially expressed between highly purified *Foxp3*^*Cre*^ and *Foxp1*^*f/f*^*Foxp3*^*Cre*^ aTreg cells. **(B)** Relationship between aTreg signature genes and Foxp1 repressed genes in rTreg cells. Two rounds of RNA sequencing were performed. **(C)** Relative mRNA levels of *E2f1*, *E2f2*, *E2f3*, *Cdk1*, *Mcm5*, and *Pik3ip1* in purified rTreg cells and aTreg cells from 6–8-week-old *Foxp3*^*Cre*^ and *Foxp1*^*f/f*^*Foxp3*^*Cre*^ mice, *n* = 3. Data in **(C)** represent at least three independent experiments. Data in **(C)** are mean ± SEM, *P < 0.05, **P < 0.01, ***P < 0.001 (two-tailed Student *t* test). Data associated with this figure can be found in the supplemental data file **(S1 Data)**. aTreg, activated Treg; *Cdk1*, cyclin-dependent kinase 1; *E2f1*, E2F transcription factor 1; *E2f2*, E2F transcription factor 2; *E2f3*, E2F transcription factor 3; Foxp1, forkhead box P1; *Mcm5*, minichromosome maintenance complex component 5; *Pik3ip1*, phosphoinositide-3-kinase interacting protein 1; rTreg, resting Treg.
(TIF)

S7 Fig Foxp1 interacts with Foxp3 in iTreg cells and Foxp1 coordinates with Foxp3 in the regulation of CTLA-4 expression in Treg cells. **(A)** Co-immunoprecipitation of Foxp1 and Foxp3 was analyzed in iTreg cells, and isotype matched antibody (IgG) and *Foxp1*^*f/f*^*Foxp3*^*Cre*^ cells were used as controls. **(B)** Flow cytometry analysis of co-regulation of CTLA-4 by Foxp1 and Foxp3. The MFI of CTLA-4 in *Foxp1*^*f/f*^*Foxp3*^*Cre*^ iTreg cells infected with control retroviruses (RV-ctrl), retroviruses expressing Foxp1A (RV-Foxp1A), retroviruses expressing Foxp3 (RV-Foxp3), or retroviruses expressing both RV-Foxp1A and RV-Foxp3 (RV-Foxp1A+Foxp3) are shown. WT iTreg cells infected with control retroviruses were used as a control. Data in **(A** and **B)** are representative of two independent experiments. Data associated with this figure can be found in the supplemental data file **(S1 Data)**. CTLA-4, cytotoxic T-lymphocyte-associated protein 4; Foxp1, forkhead box P1; Foxp3, forkhead box P3; iTreg, induced Treg cells; MFI, mean fluorescence intensity; Treg, regulatory T; WT, wild-type.
(TIF)

S1 Table Related to Fig 7. Differentially expressed genes between *Foxp3*^*Cre*^ and *Foxp1*^*f/f*^*Foxp3*^*Cre*^ rTreg cells. rTreg, resting Treg.
(XLSX)

S2 Table Related to Fig 7. Differentially expressed genes between *Foxp3*^*Cre*^ and *Foxp1*^*f/f*^*Foxp3*^*Cre*^ aTreg cells. aTreg, activated Treg.
(XLSX)

S3 Table Related to Fig 7. aTreg signature genes. aTreg, activated Treg.
(XLSX)

S4 Table Related to Fig 8. Common target genes of Foxp1 and Foxp3 in rTreg cells. Foxp1, forkhead box P1; Foxp3, forkhead box P3; rTreg, resting Treg.
(XLSX)

S1 Data Numerical values of presented diagrams. (XLS)

## Abbreviations

Aiolos/Ikzf3: IKAROS family zinc finger 3
aTreg: activated Treg
Bcl11b: B cell leukemia/lymphoma 11B
BFA: Brefeldin A
BP: biological process
c-Rel: reticuloendotheliosis oncogene
CD: cluster of differentiation
Cdk1: cyclin-dependent kinase 1
CFA: Complete Freund’s Adjuvant
ChIP: chromatin immunoprecipitation
Cnot3: CCR4-NOT transcription complex, subunit 3
CNS: conserved noncoding sequence
CNS2: conserved noncoding sequence 2
CREB: cyclic-AMP response element binding protein
CTLA-4: cytotoxic T-lymphocyte-associated protein 4
d: day
DMEM: Dulbecco’s Modified Eagle Medium
DP: double-positive
DSS: dextran sulfate sodium
EAE: experimental autoimmune encephalomyelitis
Ets: E26 avian leukemia oncogene
Ets-1: E26 avian leukemia oncogene 1, 5' domain
E2f: E2 promoter binding factor
FOX: forkhead box
Foxo: forkhead box O
Foxo1: forkhead box O1
Foxp: P subfamily members of the FOX transcription factor family
Foxp1: forkhead box P1
Foxp4: forkhead box P4
FPKM: fragments per kilobase of transcripts per million fragments mapped
Gata3: GATA binding protein 3
GO: Gene Ontology
GSEA: gene set enrichment analysis
IFNγ: interferon gamma
Ikaros/Ikzf1: IKAROS family zinc finger 1
IL: interleukin
IRF4: interferon regulatory factor 4
iTreg: induced Treg cells
KEGG: Kyoto Encyclopedia of Genes and Genomes
Ki-67: antigen identified by monoclonal antibody Ki 67
LoxP: locus of crossing over (x), P1
LP: lamina propria
Myb: myeloblastosis oncogene
NFAT: nuclear factor of activated T cells
NFATc2: nuclear factor of activated T cells, cytoplasmic, calcineurin-dependent 2
NF-κB: activation of nuclear factor κB
NF-κB c-Rel: the c-Rel subunit of NF-κB
Pik3ip1: phosphoinositide-3-kinase interacting protein 1
PT: pertussis toxin
pTreg: Treg cell generated in the periphery
Rag1: recombination activating 1
RNA-seq: RNA sequencing
rTreg: resting Treg
Runx1: runt related transcription factor 1
RV-Foxp3: Foxp3 retroviral expression vector
Satb1: special AT-rich sequence binding protein 1
Smad3: SMAD family member 3
STAT3: signal transducer and activator of transcription
STAT5: signal transducer and activator of transcription 5
TCR: T-cell receptor
T_FH_: follicular helper T cell
TGF-β: transforming growth factor, beta
T_H_1: T helper 1
T_H_17: T helper 17
Treg: regulatory T
TSS: transcription start site
tTreg: thymus-derived Treg
WT: wild-type

## References

[1] Piccirillo CA, d'Hennezel E, Sgouroudis E, Yurchenko E (2008) CD4+Foxp3+ regulatory T cells in the control of autoimmunity: in vivo veritas. Curr Opin Immunol 20: 655–662. 10.1016/j.coi.2008.09.006 18926906

[2] Sakaguchi S, Yamaguchi T, Nomura T, Ono M (2008) Regulatory T cells and immune tolerance. Cell 133: 775–787. 10.1016/j.cell.2008.05.009 18510923

[3] Smigiel KS, Richards E, Srivastava S, Thomas KR, Dudda JC, et al (2014) CCR7 provides localized access to IL-2 and defines homeostatically distinct regulatory T cell subsets. J Exp Med 211: 121–136. 10.1084/jem.20131142 24378538 PMC3892972

[4] Luo CT, Liao W, Dadi S, Toure A, Li MO (2016) Graded Foxo1 activity in Treg cells differentiates tumour immunity from spontaneous autoimmunity. Nature 529: 532–536. 10.1038/nature16486 26789248 PMC4978705

[5] Huehn J, Siegmund K, Lehmann JC, Siewert C, Haubold U, et al (2004) Developmental stage, phenotype, and migration distinguish naive- and effector/memory-like CD4+ regulatory T cells. J Exp Med 199: 303–313. 10.1084/jem.20031562 14757740 PMC2211798

[6] Dias S, D'Amico A, Cretney E, Liao Y, Tellier J, et al (2017) Effector Regulatory T Cell Differentiation and Immune Homeostasis Depend on the Transcription Factor Myb. Immunity 46: 78–91. 10.1016/j.immuni.2016.12.017 28099866

[7] Cretney E, Xin A, Shi W, Minnich M, Masson F, et al (2011) The transcription factors Blimp-1 and IRF4 jointly control the differentiation and function of effector regulatory T cells. Nat Immunol 12: 304–311. 10.1038/ni.2006 21378976

[8] Grinberg-Bleyer Y, Oh H, Desrichard A, Bhatt DM, Caron R, et al (2017) NF-kappaB c-Rel Is Crucial for the Regulatory T Cell Immune Checkpoint in Cancer. Cell 170: 1096–1108 e1013. 10.1016/j.cell.2017.08.004 28886380 PMC5633372

[9] Josefowicz SZ, Lu LF, Rudensky AY (2012) Regulatory T cells: mechanisms of differentiation and function. Annu Rev Immunol 30: 531–564. 10.1146/annurev.immunol.25.022106.141623 22224781 PMC6066374

[10] Fontenot JD, Gavin MA, Rudensky AY (2003) Foxp3 programs the development and function of CD4+CD25+ regulatory T cells. Nat Immunol 4: 330–336. 10.1038/ni904 12612578

[11] Khattri R, Cox T, Yasayko SA, Ramsdell F (2003) An essential role for Scurfin in CD4+CD25+ T regulatory cells. Nat Immunol 4: 337–342. 10.1038/ni909 12612581

[12] Gambineri E, Torgerson TR, Ochs HD (2003) Immune dysregulation, polyendocrinopathy, enteropathy, and X-linked inheritance (IPEX), a syndrome of systemic autoimmunity caused by mutations of FOXP3, a critical regulator of T-cell homeostasis. Curr Opin Rheumatol 15: 430–435. 12819471 10.1097/00002281-200307000-00010

[13] Williams LM, Rudensky AY (2007) Maintenance of the Foxp3-dependent developmental program in mature regulatory T cells requires continued expression of Foxp3. Nat Immunol 8: 277–284. 10.1038/ni1437 17220892

[14] Wan YY, Flavell RA (2007) Regulatory T-cell functions are subverted and converted owing to attenuated Foxp3 expression. Nature 445: 766–770. 10.1038/nature05479 17220876

[15] Li X, Liang Y, LeBlanc M, Benner C, Zheng Y (2014) Function of a Foxp3 cis-element in protecting regulatory T cell identity. Cell 158: 734–748. 10.1016/j.cell.2014.07.030 25126782 PMC4151505

[16] Feng Y, Arvey A, Chinen T, van der Veeken J, Gasteiger G, et al (2014) Control of the inheritance of regulatory T cell identity by a cis element in the Foxp3 locus. Cell 158: 749–763. 10.1016/j.cell.2014.07.031 25126783 PMC4151558

[17] Wang Y, Su MA, Wan YY (2011) An essential role of the transcription factor GATA-3 for the function of regulatory T cells. Immunity 35: 337–348. 10.1016/j.immuni.2011.08.012 21924928 PMC3182399

[18] Wohlfert EA, Grainger JR, Bouladoux N, Konkel JE, Oldenhove G, et al (2011) GATA3 controls Foxp3(+) regulatory T cell fate during inflammation in mice. J Clin Invest 121: 4503–4515. 10.1172/JCI57456 21965331 PMC3204837

[19] Mouly E, Chemin K, Nguyen HV, Chopin M, Mesnard L, et al (2010) The Ets-1 transcription factor controls the development and function of natural regulatory T cells. J Exp Med 207: 2113–2125. 10.1084/jem.20092153 20855499 PMC2947068

[20] Kim HP, Leonard WJ (2007) CREB/ATF-dependent T cell receptor-induced FoxP3 gene expression: a role for DNA methylation. J Exp Med 204: 1543–1551. 10.1084/jem.20070109 17591856 PMC2118651

[21] Ouyang W, Beckett O, Ma Q, Paik JH, DePinho RA, et al (2010) Foxo proteins cooperatively control the differentiation of Foxp3+ regulatory T cells. Nat Immunol 11: 618–627. 10.1038/ni.1884 20467422

[22] Tone Y, Furuuchi K, Kojima Y, Tykocinski ML, Greene MI, et al (2008) Smad3 and NFAT cooperate to induce Foxp3 expression through its enhancer. Nat Immunol 9: 194–202. 10.1038/ni1549 18157133

[23] Yao Z, Kanno Y, Kerenyi M, Stephens G, Durant L, et al (2007) Nonredundant roles for Stat5a/b in directly regulating Foxp3. Blood 109: 4368–4375. 10.1182/blood-2006-11-055756 17227828 PMC1885496

[24] Kitoh A, Ono M, Naoe Y, Ohkura N, Yamaguchi T, et al (2009) Indispensable role of the Runx1-Cbfbeta transcription complex for in vivo-suppressive function of FoxP3+ regulatory T cells. Immunity 31: 609–620. 10.1016/j.immuni.2009.09.003 19800266

[25] Ruan Q, Kameswaran V, Tone Y, Li L, Liou HC, et al (2009) Development of Foxp3(+) regulatory t cells is driven by the c-Rel enhanceosome. Immunity 31: 932–940. 10.1016/j.immuni.2009.10.006 20064450 PMC2807990

[26] Kitagawa Y, Ohkura N, Kidani Y, Vandenbon A, Hirota K, et al (2017) Guidance of regulatory T cell development by Satb1-dependent super-enhancer establishment. Nat Immunol 18: 173–183. 10.1038/ni.3646 27992401 PMC5582804

[27] Zheng Y, Josefowicz S, Chaudhry A, Peng XP, Forbush K, et al (2010) Role of conserved non-coding DNA elements in the Foxp3 gene in regulatory T-cell fate. Nature 463: 808–812. 10.1038/nature08750 20072126 PMC2884187

[28] Rudra D, deRoos P, Chaudhry A, Niec RE, Arvey A, et al (2012) Transcription factor Foxp3 and its protein partners form a complex regulatory network. Nat Immunol 13: 1010–1019. 10.1038/ni.2402 22922362 PMC3448012

[29] Hu H, Wang B, Borde M, Nardone J, Maika S, et al (2006) Foxp1 is an essential transcriptional regulator of B cell development. Nat Immunol 7: 819–826. 10.1038/ni1358 16819554

[30] Feng X, Ippolito GC, Tian L, Wiehagen K, Oh S, et al (2010) Foxp1 is an essential transcriptional regulator for the generation of quiescent naive T cells during thymocyte development. Blood 115: 510–518. 10.1182/blood-2009-07-232694 19965654 PMC2810984

[31] Feng X, Wang H, Takata H, Day TJ, Willen J, et al (2011) Transcription factor Foxp1 exerts essential cell-intrinsic regulation of the quiescence of naive T cells. Nat Immunol 12: 544–550. 10.1038/ni.2034 21532575 PMC3631322

[32] Wang H, Geng J, Wen X, Bi E, Kossenkov AV, et al (2014) The transcription factor Foxp1 is a critical negative regulator of the differentiation of follicular helper T cells. Nat Immunol 15: 667–675. 10.1038/ni.2890 24859450 PMC4142638

[33] Li B, Samanta A, Song X, Iacono KT, Brennan P, et al (2007) FOXP3 is a homo-oligomer and a component of a supramolecular regulatory complex disabled in the human XLAAD/IPEX autoimmune disease. Int Immunol 19: 825–835. 10.1093/intimm/dxm043 17586580

[34] Rubtsov YP, Rasmussen JP, Chi EY, Fontenot J, Castelli L, et al (2008) Regulatory T cell-derived interleukin-10 limits inflammation at environmental interfaces. Immunity 28: 546–558. 10.1016/j.immuni.2008.02.017 18387831

[35] Scholzen T, Gerdes J (2000) The Ki-67 protein: from the known and the unknown. J Cell Physiol 182: 311–322. 10.1002/(SICI)1097-4652(200003)182:3<311::AID-JCP1>3.0.CO;2-9 10653597

[36] Korn T, Bettelli E, Oukka M, Kuchroo VK (2009) IL-17 and Th17 Cells. Annu Rev Immunol 27: 485–517. 10.1146/annurev.immunol.021908.132710 19132915

[37] Chen W, Jin W, Hardegen N, Lei KJ, Li L, et al (2003) Conversion of peripheral CD4+CD25- naive T cells to CD4+CD25+ regulatory T cells by TGF-beta induction of transcription factor Foxp3. J Exp Med 198: 1875–1886. 10.1084/jem.20030152 14676299 PMC2194145

[38] Wei H, Geng J, Shi B, Liu Z, Wang YH, et al (2016) Cutting Edge: Foxp1 Controls Naive CD8+ T Cell Quiescence by Simultaneously Repressing Key Pathways in Cellular Metabolism and Cell Cycle Progression. J Immunol 196: 3537–3541. 10.4049/jimmunol.1501896 27001958 PMC4868629

[39] Hill JA, Feuerer M, Tash K, Haxhinasto S, Perez J, et al (2007) Foxp3 transcription-factor-dependent and -independent regulation of the regulatory T cell transcriptional signature. Immunity 27: 786–800. 10.1016/j.immuni.2007.09.010 18024188

[40] Wang B, Lin D, Li C, Tucker P (2003) Multiple domains define the expression and regulatory properties of Foxp1 forkhead transcriptional repressors. J Biol Chem 278: 24259–24268. 10.1074/jbc.M207174200 12692134

[41] Zheng Y, Josefowicz SZ, Kas A, Chu TT, Gavin MA, et al (2007) Genome-wide analysis of Foxp3 target genes in developing and mature regulatory T cells. Nature 445: 936–940. 10.1038/nature05563 17237761

[42] Shi B, Geng J, Wang YH, Wei H, Walters B, et al (2018) Foxp1 Negatively Regulates T Follicular Helper Cell Differentiation and Germinal Center Responses by Controlling Cell Migration and CTLA-4. J Immunol 200: 586–594. 10.4049/jimmunol.1701000 29212910 PMC5891213

[43] Wang Y, Morrisey E (2010) Regulation of cardiomyocyte proliferation by Foxp1. Cell Cycle 9: 4251–4252. 10.4161/cc.9.21.13924 21051948

[44] Zhai L, Zhao Y, Ye S, Huang H, Tian Y, et al (2011) Expression of PIK3CA and FOXP1 in gastric and intestinal non-Hodgkin's lymphoma of mucosa-associated lymphoid tissue type. Tumour Biol 32: 913–920. 10.1007/s13277-011-0192-3 21660567

[45] Bacon C, Schneider M, Le Magueresse C, Froehlich H, Sticht C, et al (2015) Brain-specific Foxp1 deletion impairs neuronal development and causes autistic-like behaviour. Mol Psychiatry 20: 632–639. 10.1038/mp.2014.116 25266127 PMC4419151

[46] Shu W, Lu MM, Zhang Y, Tucker PW, Zhou D, et al (2007) Foxp2 and Foxp1 cooperatively regulate lung and esophagus development. Development 134: 1991–2000. 10.1242/dev.02846 17428829

[47] Wang B, Weidenfeld J, Lu MM, Maika S, Kuziel WA, et al (2004) Foxp1 regulates cardiac outflow tract, endocardial cushion morphogenesis and myocyte proliferation and maturation. Development 131: 4477–4487. 10.1242/dev.01287 15342473

[48] Ghosh S, Roy-Chowdhuri S, Kang K, Im SH, Rudra D (2018) The transcription factor Foxp1 preserves integrity of an active Foxp3 locus in extrathymic Treg cells. Nat Commun 9: 4473 10.1038/s41467-018-07018-y 30367168 PMC6203760

[49] Konopacki C, Pritykin Y, Rubtsov Y, Leslie CS, Rudensky AY (2019) Transcription factor Foxp1 regulates Foxp3 chromatin binding and coordinates regulatory T cell function. Nat Immunol 20: 232–242. 10.1038/s41590-018-0291-z 30643266 PMC7534899

[50] Bruno L, Mazzarella L, Hoogenkamp M, Hertweck A, Cobb BS, et al (2009) Runx proteins regulate Foxp3 expression. J Exp Med 206: 2329–2337. 10.1084/jem.20090226 19841090 PMC2768863

[51] Wing K, Onishi Y, Prieto-Martin P, Yamaguchi T, Miyara M, et al (2008) CTLA-4 control over Foxp3+ regulatory T cell function. Science 322: 271–275. 10.1126/science.1160062 18845758

[52] Stromnes IM, Goverman JM (2006) Active induction of experimental allergic encephalomyelitis. Nat Protoc 1: 1810–1819. 10.1038/nprot.2006.285 17487163

[53] Law CW, Chen Y, Shi W, Smyth GK (2014) voom: Precision weights unlock linear model analysis tools for RNA-seq read counts. Genome Biol 15: R29 10.1186/gb-2014-15-2-r29 24485249 PMC4053721

[54] Liu R, Holik AZ, Su S, Jansz N, Chen K, et al (2015) Why weight? Modelling sample and observational level variability improves power in RNA-seq analyses. Nucleic Acids Res 43: e97 10.1093/nar/gkv412 25925576 PMC4551905

