# Supplementary figures and images for: Foxp1 is critical for the maintenance of regulatory T-cell homeostasis and suppressive function
Source: PLoS Biol. 2019 May 24;17(5):e3000270. doi: 10.1371/journal.pbio.3000270 (PMC6534289; doi:10.1371/journal.pbio.3000270)

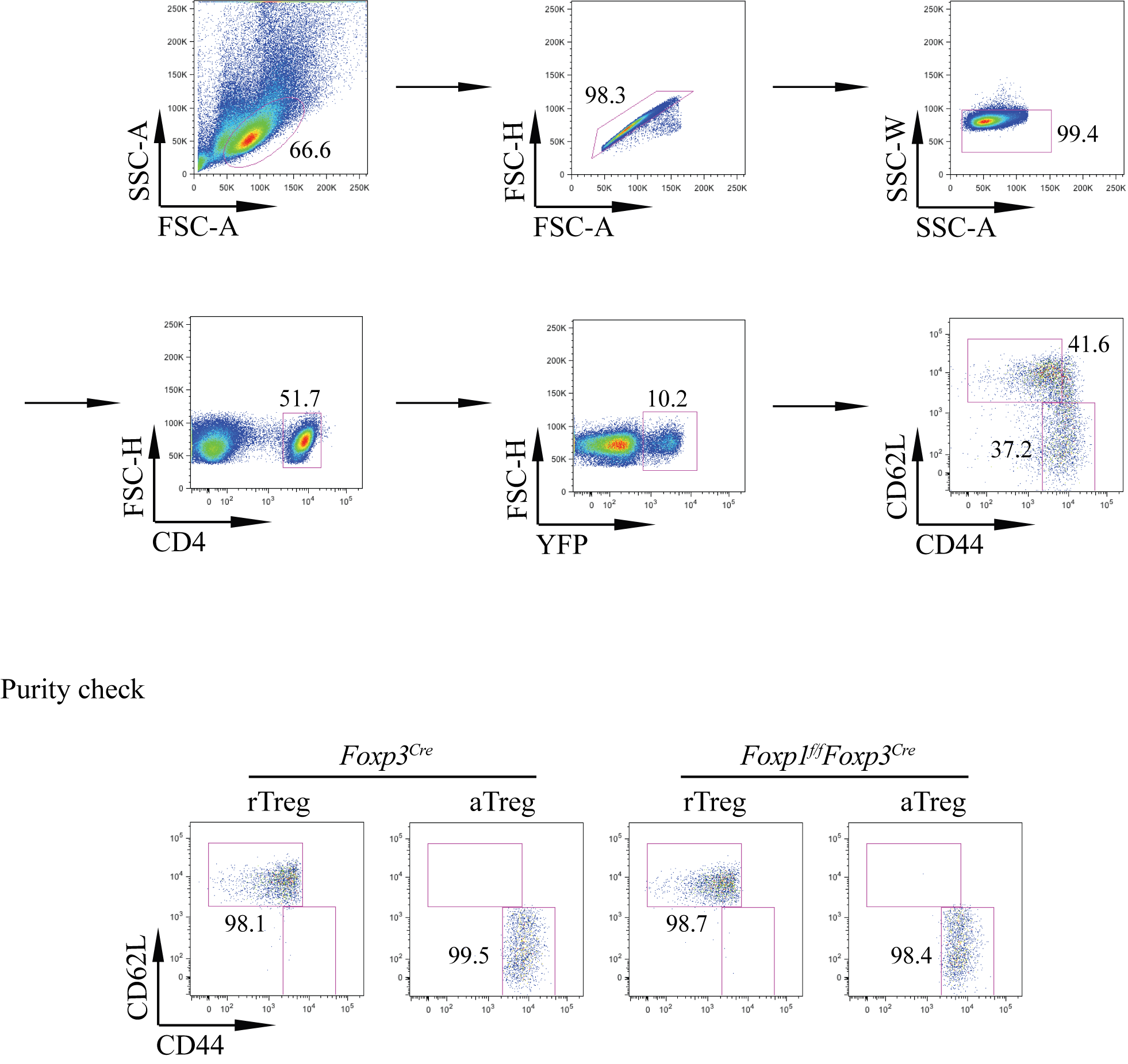

Supplement: S1 Fig — The gating strategy of CD44lowCD62Lhigh rTreg and CD44highCD62Llow aTreg (upper panel), and the purity check after cell sorting of CD44lowCD62Lhigh rTreg and CD44highCD62Llow aTreg in Foxp3Cre and Foxp1f/fFoxp3Cre mice (lower panel). aTreg, activated Treg; rTreg, resting Treg. (TIF) [file pbio.3000270.s001.tif]

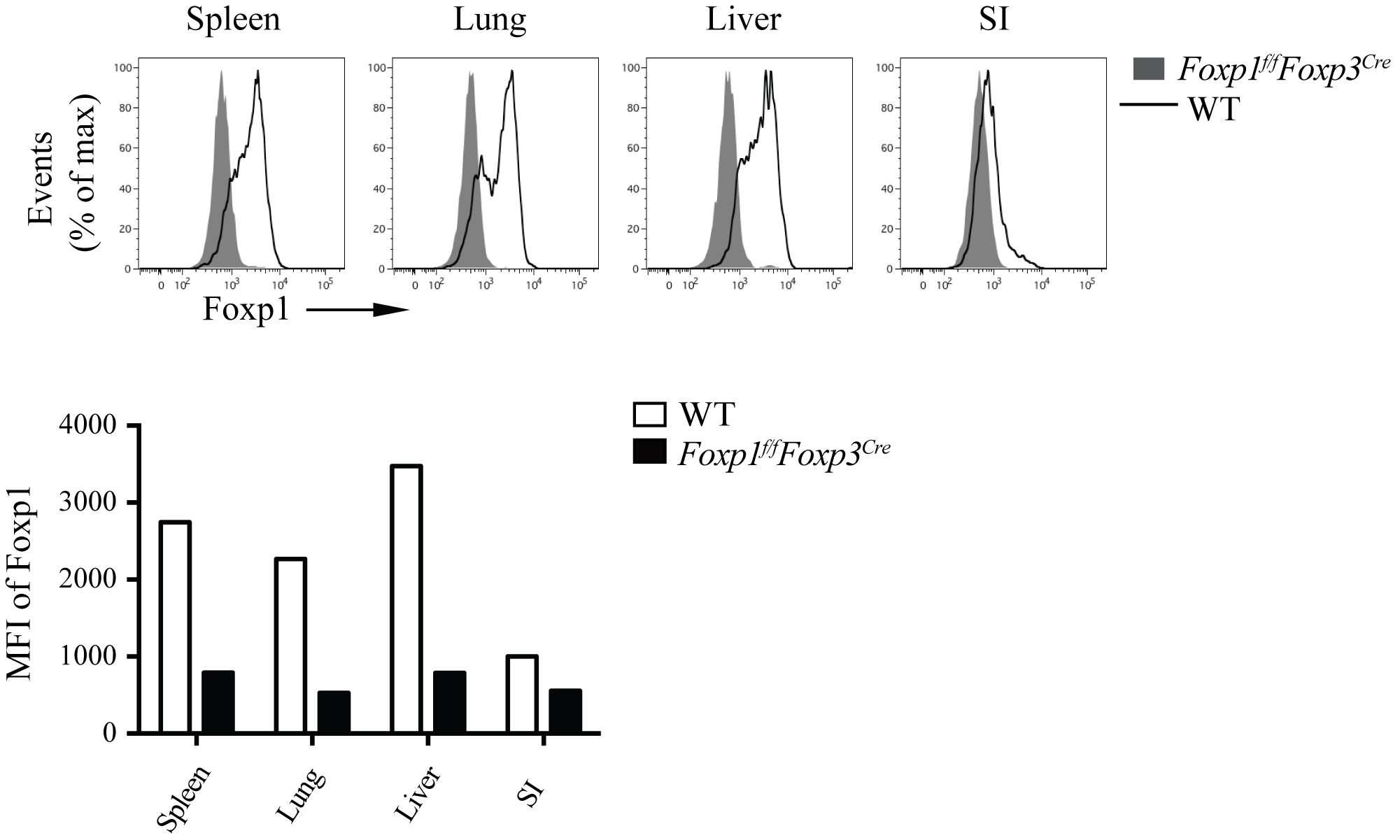

Supplement: S2 Fig — Intracellular staining of Foxp1 in Treg cells in spleens, lungs, livers, small intestines (SI) from Foxp3Cre mice (WT) (upper panel), and the corresponding MFI of Foxp1 (lower panel). Foxp1-deficient Treg cells were used as staining controls. Data are representative of two independent experiments. Data associated with this figure can be found in the supplemental data file (S1 Data). Foxp1, forkhead box P1; MFI, mean fluorescence intensity; SI, small intestine; Treg, regulatory T; WT, wild-type. (TIF) [file pbio.3000270.s002.tif]

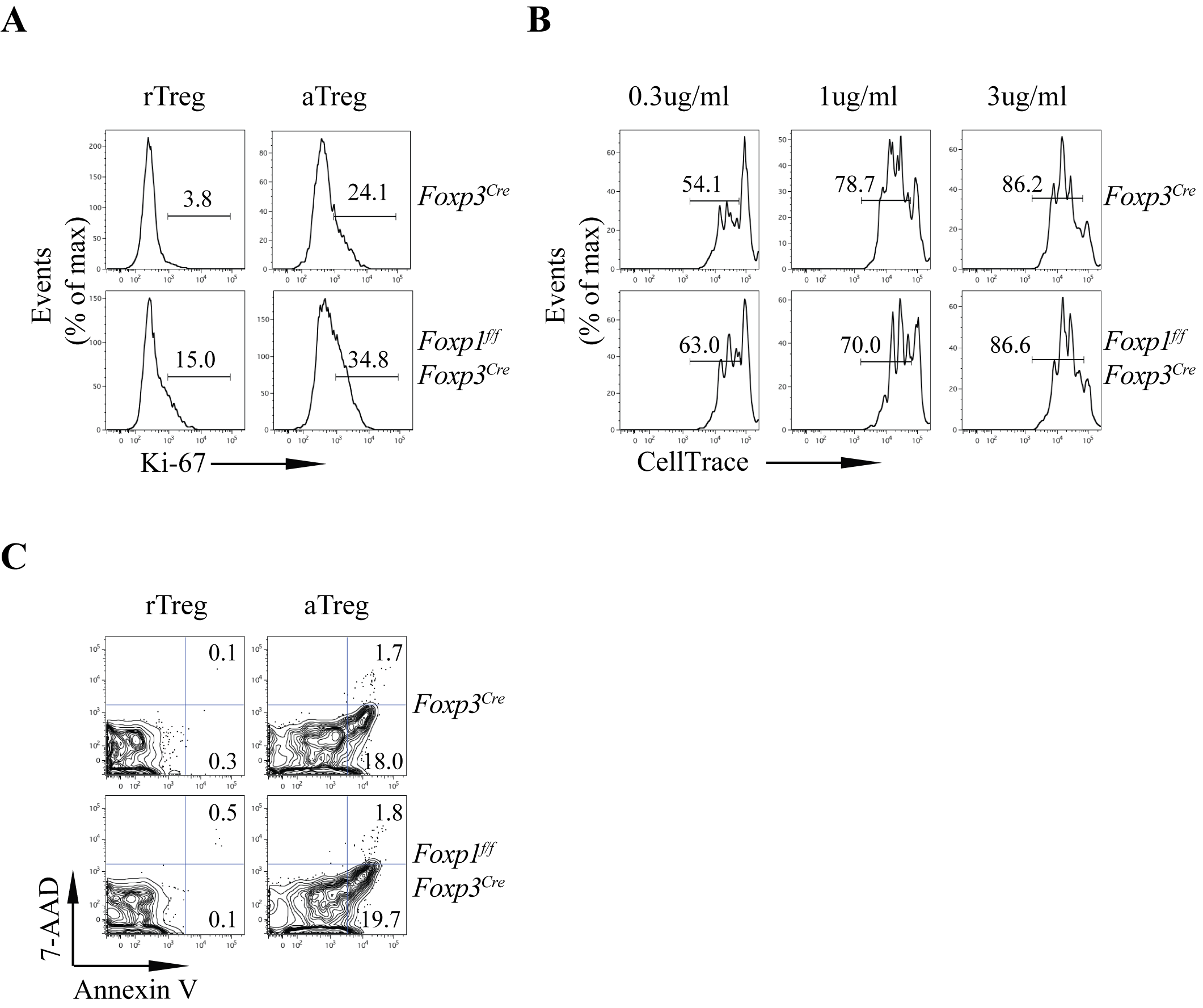

Supplement: S3 Fig — (A) Flow cytometry analysis of Ki-67 expression in rTreg and aTreg cells in spleens from 3-week-old Foxp3Cre and Foxp1f/fFoxp3Cre mice; numbers above bracketed lines indicate percent Ki-67+ Treg cells. (B) Proliferation of aTreg cells cultured with different concentrations of anti-CD3 and anti-CD28 antibodies in the presence of 200 U/mL IL-2 for 3 days, as shown by dilution of CellTrace; numbers above bracketed lines indicate percent proliferating cells. (C) Annexin V and 7-AAD staining in rTreg and aTreg cells in spleens from 6-week-old Foxp3Cre and Foxp1f/fFoxp3Cre mice; numbers adjacent to the outlined area represent the percentage of gated cells. Data are representative of two independent experiments. aTreg, activated Treg; IL, interleukin; Ki-67, antigen identified by monoclonal antibody Ki 67; rTreg, resting Treg. (TIF) [file pbio.3000270.s003.tif]

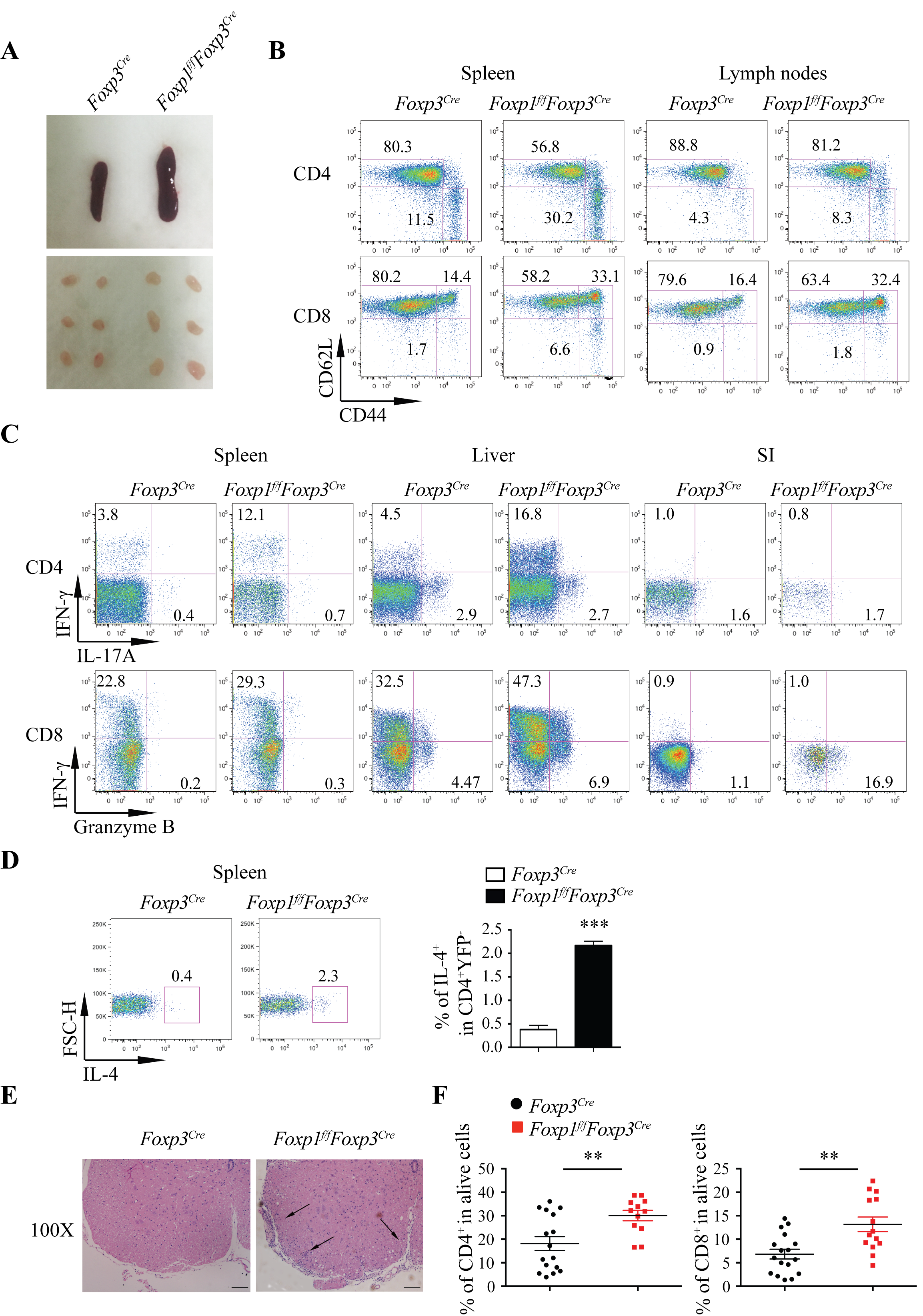

Supplement: S4 Fig — (A) Representative picture of spleens and lymph nodes from 8-week-old sex- and age-matched Foxp3Cre and Foxp1f/fFoxp3Cre mice. (B) Flow cytometry analysis of CD44 and CD62L expression in conventional CD4+ T cells (YFP−CD4+) and CD8+ T cells in spleens and lymph nodes from 8-week-old Foxp3Cre and Foxp1f/fFoxp3Cre mice; numbers adjacent to the outlined area represent the percentage of gated cells. (C) Intracellular staining of cytokines in conventional CD4+ and CD8+ T cells in spleens, livers, and small intestines (SI) from 8-week-old Foxp3Cre and Foxp1f/fFoxp3Cre mice; numbers adjacent to the outlined area represent the percentage of gated cells. (D) Intracellular staining of IL-4 in YFP−CD4+ T cells in the spleens from 28-week-old Foxp3Cre and Foxp1f/fFoxp3Cre mice (left panel) and the corresponding statistics (right panel) (n = 3). Numbers adjacent to the outlined area indicate the percentage of IL-4+ cells in CD4+ T cells. (E) Hematoxylin and eosin staining of spinal cord sections (×100, scale bars, 100 μm) from Foxp3Cre and Foxp1f/fFoxp3Cre mice induced with EAE by immunization with MOG peptide and PT. (F) Quantification of CD4+ and CD8+ T-cell frequencies in alive cells from brains and spinal cords of Foxp3Cre or Foxp1f/fFoxp3Cre mice (n = 12–17). Data in (A-C, F) are representative of at least three independent experiments. Data in (D and E) are representative of two independent experiments. Data in (D: right panel, F) are mean ± SEM, **P < 0.01, ***P < 0.001 (two-tailed Student t test). Data associated with this figure can be found in the supplemental data file (S1 Data). CD, cluster of differentiation; EAE, experimental autoimmune encephalomyelitis; IL, interleukin; MOG, myelin oligodendrocyte glycoprotein; PT, pertussis toxin; SI, small intestine. (TIF) [file pbio.3000270.s004.tif]

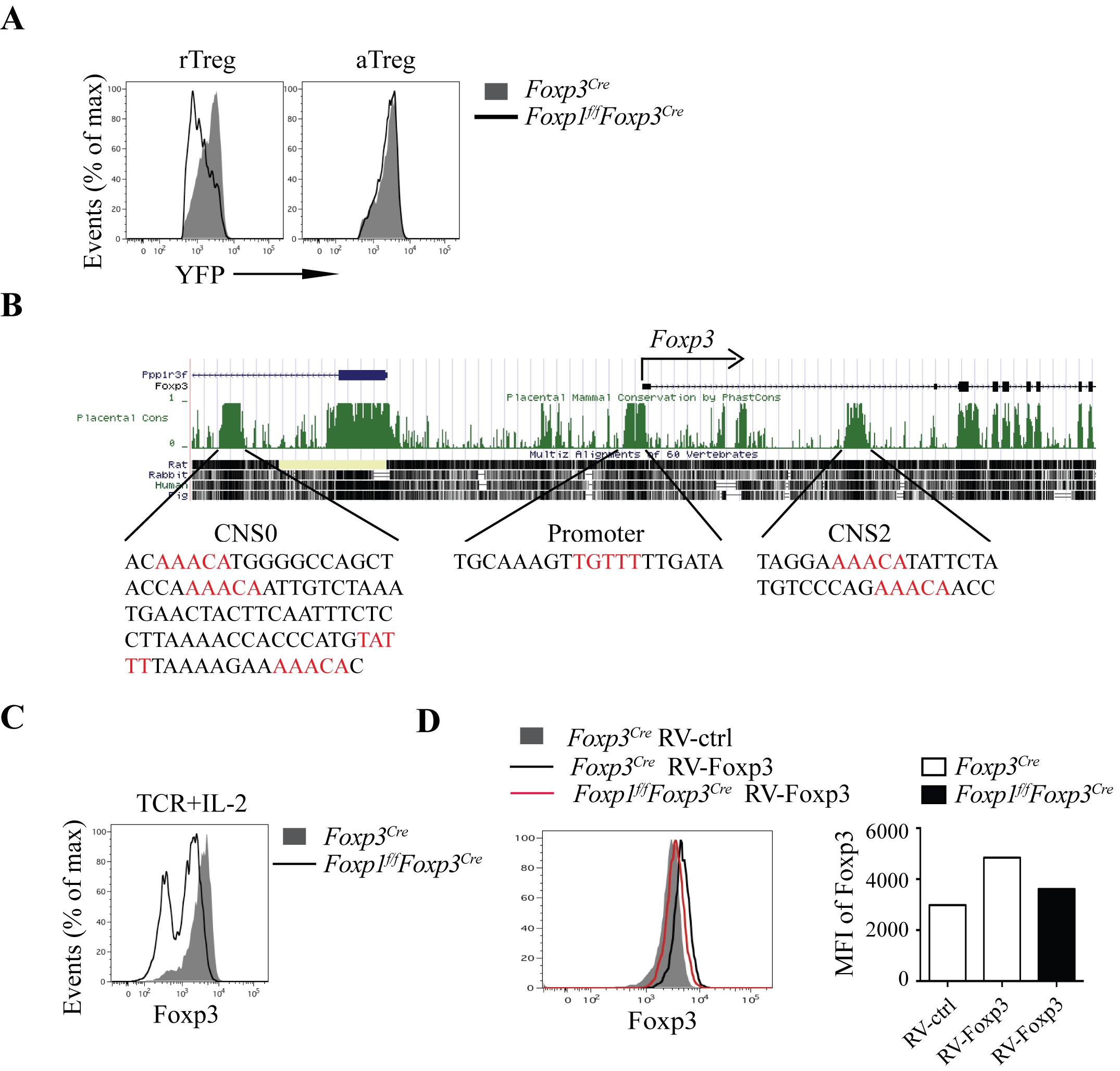

Supplement: S5 Fig — (A) Flow cytometry analysis of YFP expression in rTreg and aTreg cells from the spleens of Foxp3Cre and Foxp1f/fFoxp3Cre mice. (B) Predicted conserved forkhead-binding sites (highlighted in red) in the CNS0, promoter, and CNS2 region of Foxp3, respectively. (C) Sorted YFP+ Foxp3Cre and Foxp1f/fFoxp3Cre iTreg cells were stimulated with anti-CD3 and -CD28 beads for 3 days, and intracellular staining of Foxp3 in Foxp3Cre and Foxp1f/fFoxp3Cre iTreg cells were analyzed by flow cytometry. (D) Intracellular staining of Foxp3 in Foxp3Cre and Foxp1f/fFoxp3Cre iTreg cells infected with control retroviruses (RV-ctrl) or retroviruses expressing Foxp3 (RV-Foxp3) (left panel), and the corresponding MFI of Foxp3 (right panel). Data in (A, C) are representative of at least three independent experiments. Data in (D) are representative of two independent experiments. Data associated with this figure can be found in the supplemental data file (S1 Data). aTreg, activated Treg; CNS, conserved noncoding sequence; Foxp1, forkhead box P1; Foxp3, forkhead box P3; iTreg, induced Treg cells; MFI, mean fluorescence intensity; rTreg, resting Treg; YFP, yellow fluorescent protein. (TIF) [file pbio.3000270.s005.tif]

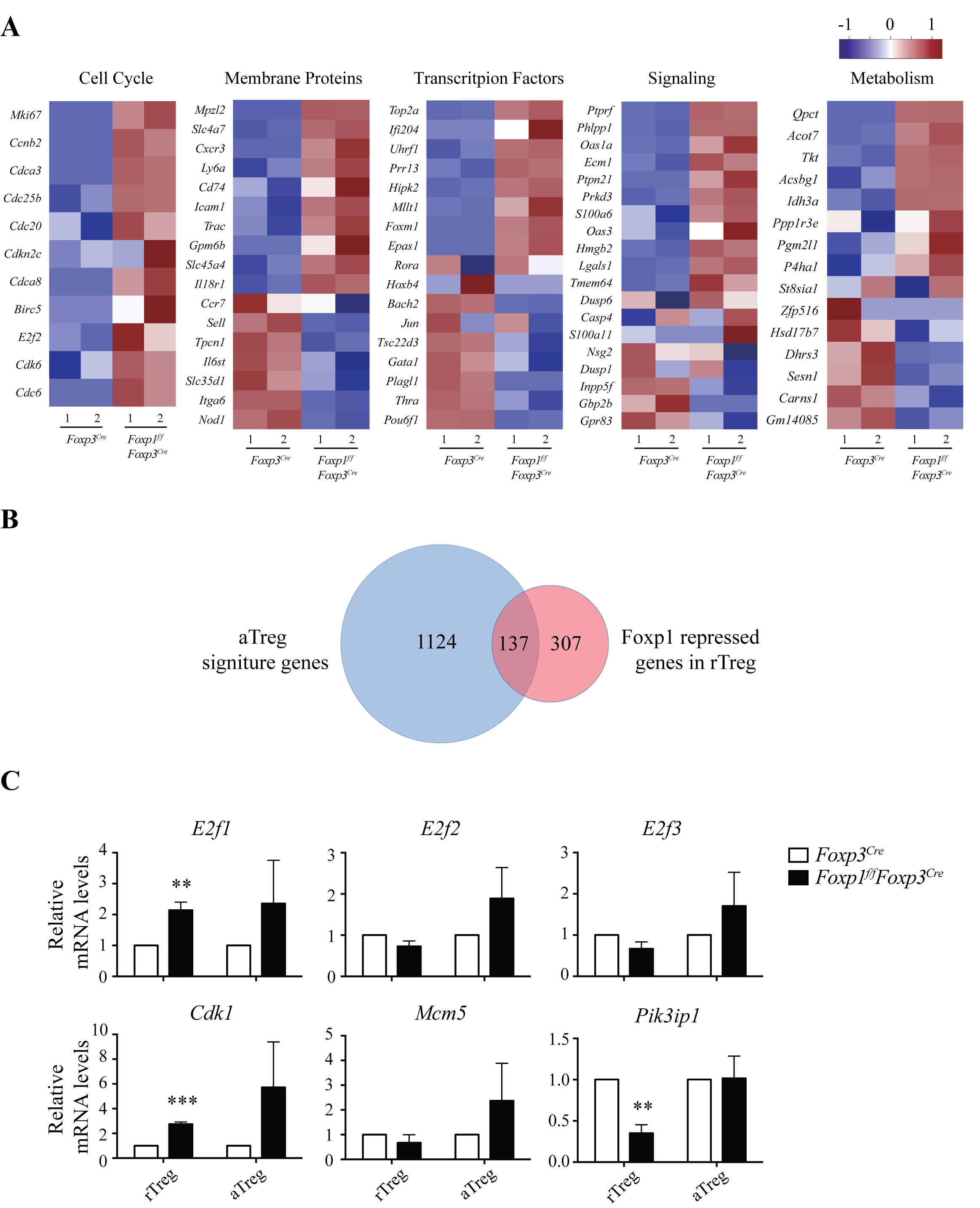

Supplement: S6 Fig — (A) Representative genes differentially expressed between highly purified Foxp3Cre and Foxp1f/fFoxp3Cre aTreg cells. (B) Relationship between aTreg signature genes and Foxp1 repressed genes in rTreg cells. Two rounds of RNA sequencing were performed. (C) Relative mRNA levels of E2f1, E2f2, E2f3, Cdk1, Mcm5, and Pik3ip1 in purified rTreg cells and aTreg cells from 6–8-week-old Foxp3Cre and Foxp1f/fFoxp3Cre mice, n = 3. Data in (C) represent at least three independent experiments. Data in (C) are mean ± SEM, *P < 0.05, **P < 0.01, ***P < 0.001 (two-tailed Student t test). Data associated with this figure can be found in the supplemental data file (S1 Data). aTreg, activated Treg; Cdk1, cyclin-dependent kinase 1; E2f1, E2F transcription factor 1; E2f2, E2F transcription factor 2; E2f3, E2F transcription factor 3; Foxp1, forkhead box P1; Mcm5, minichromosome maintenance complex component 5; Pik3ip1, phosphoinositide-3-kinase interacting protein 1; rTreg, resting Treg. (TIF) [file pbio.3000270.s006.tif]

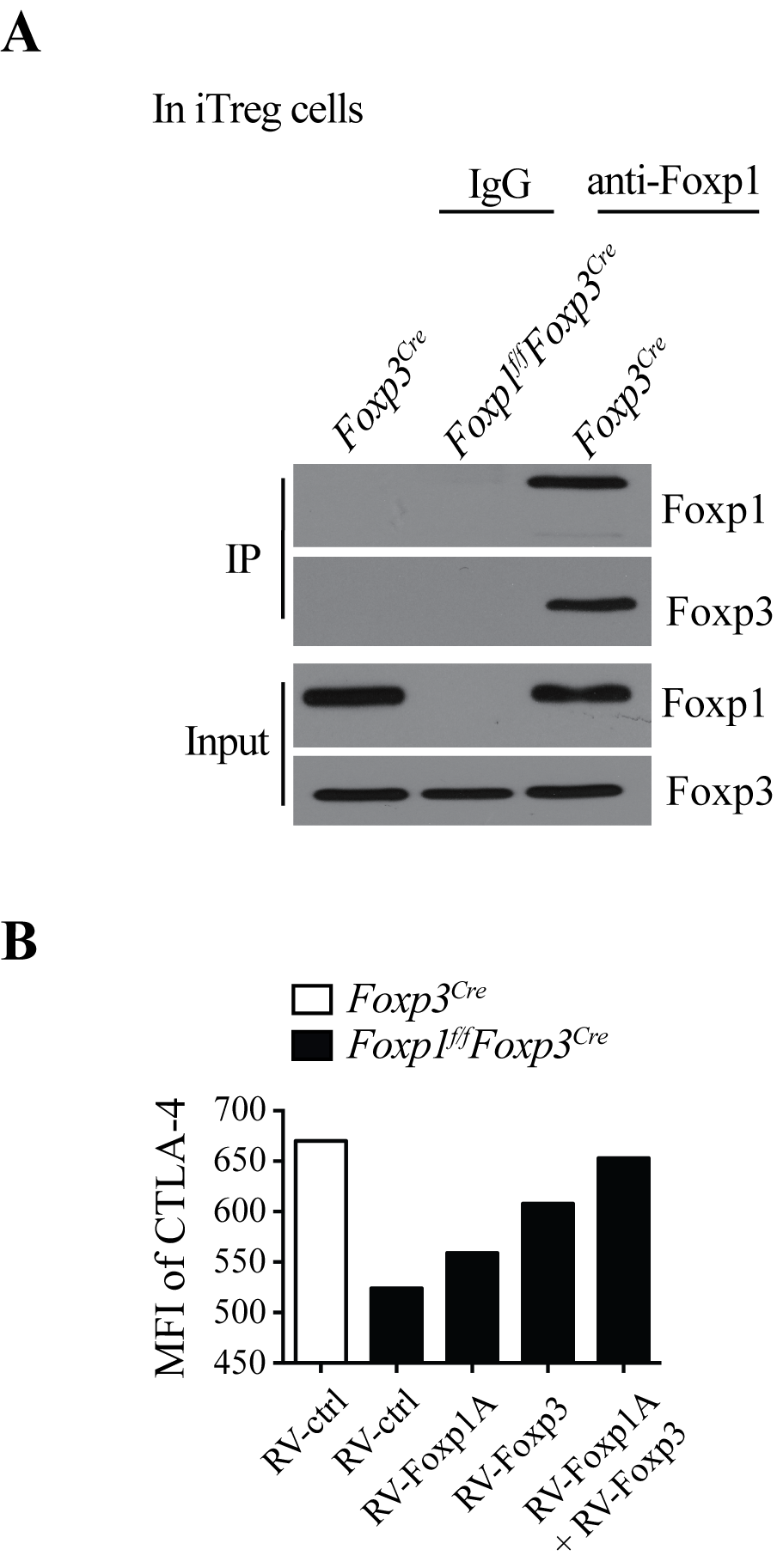

Supplement: S7 Fig — (A) Co-immunoprecipitation of Foxp1 and Foxp3 was analyzed in iTreg cells, and isotype matched antibody (IgG) and Foxp1f/fFoxp3Cre cells were used as controls. (B) Flow cytometry analysis of co-regulation of CTLA-4 by Foxp1 and Foxp3. The MFI of CTLA-4 in Foxp1f/fFoxp3Cre iTreg cells infected with control retroviruses (RV-ctrl), retroviruses expressing Foxp1A (RV-Foxp1A), retroviruses expressing Foxp3 (RV-Foxp3), or retroviruses expressing both RV-Foxp1A and RV-Foxp3 (RV-Foxp1A+Foxp3) are shown. WT iTreg cells infected with control retroviruses were used as a control. Data in (A and B) are representative of two independent experiments. Data associated with this figure can be found in the supplemental data file (S1 Data). CTLA-4, cytotoxic T-lymphocyte-associated protein 4; Foxp1, forkhead box P1; Foxp3, forkhead box P3; iTreg, induced Treg cells; MFI, mean fluorescence intensity; Treg, regulatory T; WT, wild-type. (TIF) [file pbio.3000270.s007.tif]
